# The Multifaceted Ganzfeld at the Crossroad Between Visual Perception and Consciousness: Behavioral, Neural and Qualitative Aspects

**DOI:** 10.1162/OPMI.a.255

**Published:** 2025-11-10

**Authors:** Eleftheria Pistolas, Boris Quétard, Sucharit Katyal, Johan Wagemans

**Affiliations:** Laboratory of Experimental Psychology, Department of Brain and Cognition, University of Leuven, KU Leuven, Belgium; Leuven Brain Institute, University of Leuven, KU Leuven, Belgium; Department of Psychology, University of Copenhagen, Denmark

**Keywords:** perception, consciousness, Ganzfeld, eye-tracking, EEG, cognition

## Abstract

A Ganzfeld is a homogeneous visual field, devoid of any focal points. Such a stimulus has been used by researchers to study perceptual phenomena in the absence of changes in sensory structure. Others have used it to study altered states of consciousness (ASCs). Until now, these different facets have been studied separately with little attention for the emotional subjective experience. This study aimed to elucidate the perceptual, phenomenal, and emotional experience of the multifaceted Ganzfeld using a multi-method approach combining behavioral (eye-tracking) and neural (electroencephalography; EEG) measures, with qualitative (interviews) and quantitative (questionnaires) assessments. We show that Ganzfeld spaces induce ASCs and offer immersive, full-body experiences, including bodily effects. Our results pertaining to bodily sensations further prompted us to identify a perceptually grounded cognitive processing type with either an inward-directed or externally-directed focus. We also identified the presence of an abstract cognitive processing type characterized by an introspective focus and meditative experiences. At the behavioral level, decays were characterized by decreased eye movements. The lag in reporting decays and the subjective experience of decays point to the notion of mind blanking. At the neural level, we found increased theta activity preceding decays, further hinting at a potential interrelation between perceptual decays and mind blanking. Finally, decays were characterized by more alpha activity, a pattern often associated with attenuated sensory processing and states of reduced external engagement (Jensen & Mazaheri, 2010), such as relaxation. Our findings contribute to a more in-depth understanding of all the components contributing to the rich Ganzfeld experiences.

## INTRODUCTION

The term “Ganzfeld” originated from the word for “overall field” in German and refers to the condition of an unstructured, homogeneous visual field. Metzger was one of the first to study the Ganzfeld, in the context of Gestalt psychology (1930). As a consequence of its structureless nature, the Ganzfeld gives rise to a peculiar perceptual experience. This unstructured, homogeneous setting is typically unfamiliar to the human perceptual system, which in everyday life is continuously processing salient and interesting structures in our visual field to perceive and give meaning to the world around us. When such anchor points are not present, people still perceive structures for which there is no corresponding physical stimulation (Bexton et al., 1954; Cohen, 1957; Hochberg et al., 1951; Miller & Hall, 1962).

These emerging percepts are commonly referred to as hallucinations and can range from simple to more complex percepts (for an overview of the nature of these emerging percepts, see Pistolas & Wagemans, 2025). Further, the addition of homogeneous auditory noise—like white noise, violet noise, brown noise—results in a multimodal Ganzfeld (Schmidt et al., 2020; Shenyan et al., 2024). Aside from visual hallucinations, auditory hallucinations have been reported in previous research, both under unimodal and multimodal Ganzfeld stimulation (Schmidt et al., 2020; Wackermann et al., 2008). Ganzfeld exposure has also been associated with distorted time perception, with participants often overestimating time perception, such that time feels to pass more slowly under Ganzfeld stimulation (Glicksohn et al., 2019). Moreover, distortions in depth perception in the Ganzfeld have previously been reported from a line of research that focuses on visual perception (Gibson & Waddell, 1952), emphasizing how ambiguous and infinite depth seems in the Ganzfeld. A less frequently reported phenomenon entails bodily sensations such as lightness, imbalance, and dizziness (Cohen, 1960).

The Ganzfeld has so far gained attention from two fields of research, i.e., visual perception and altered states of consciousness. From a consciousness perspective, the key finding of previous research is the potential of the Ganzfeld to induce altered states of consciousness without the need for pharmacological agents. These ASCs were often assessed using the Altered States of Consciousness Rating Scale (11-ASC; Studerus et al., 2010). Past research that focused on the induction of altered states of consciousness has mainly used Ganzfeld goggles, made from ping-pong ball halves that are tightly fitted over the eye orbits, combined with a strong LED light source (Schmidt et al., 2020). In our study, we aimed to investigate the Ganzfeld using a Ganzfeld space that feels less restrictive and more immersive.

From a visual perception perspective, researchers have primarily focused on the decaying of color and brightness from the visual field. This decaying can take different forms. These different forms of decaying experiences of color and brightness have been described in a confusing and inconsistent way in past literature (Bolanowski & Doty, 1987; Cohen, 1960; Gur, 1989; Helson & Judd, 1932; Hochberg et al., 1951; Sippola & Devezin, 1969; Tepas, 1962). A gradual fading of color and brightness has been termed “fade-out”. A second type termed “black-out” consists of the visual field turning increasingly dark grey or black, sometimes occurring and disappearing quickly and often reported in a pulsating manner. Third, the term “blank-out” entails the sudden or gradual disappearance of color resulting in a light grey or white visual field. These terms have been used since the 1930s but have only recently been clearly defined to describe three distinct types of decaying phenomena (see Pistolas & Wagemans, 2025, for a more in-depth overview of these different decaying phenomena and the distinction between the three).

Early vision researchers studied the behavioral component of visual perception in the Ganzfeld via electrooculography (Kirkwood, 1967; Tepas, 1962). Kirkwood (1967) found a decrease in eye movements preceding decays of color and brightness in the visual field. Tepas (1962) studied vision in the homogeneous visual field by combining electrooculography with electroencephalography (EEG) registration to investigate both behavioral and neural components. They reported that decaying phenomena were strongly associated with reduced eye movements as well as with increased alpha activity in the brain. Earlier research reported a positive relation between resting state alpha activity and the susceptibility to experience decaying phenomena (Cohen & Cadwallader, 1958). Cohen (1960) found that alpha activity reverts to the initial level or higher during decay after a brief suppression of alpha activity, referring to this phenomenon as alpha rebound. Similarly, Adrian and Matthews (1934) reported alpha rebound after participants opened their eyes in a Ganzfeld following a brief period of closing the eyes. In general, the scarce Ganzfeld research in which neural components have been considered has emphasized the occurrence of alpha activity (Lehtonen & Lehtinen, 1972).

Ganzfeld research from a consciousness perspective investigated the neural components by looking into the ratio between low frequency alpha and high frequency alpha as an approximation of accelerations in alpha activity and found indications of accelerated alpha activity during Ganzfeld exposure (Wackermann et al., 2002). Pütz et al. (2006) investigated the temporal dynamics of these alpha accelerations and found maximum alpha accelerations around 20 to 10 seconds before the report of a hallucination. The authors interpreted these results as indications of an ‘activated waking state’ during Ganzfeld exposure as opposed to the hypnagogic state proposed in past research (Wackermann et al., 2002).

Moreover, the authors reported a significant decrease in alpha power (specifically the lower alpha range, i.e., 7–10 Hz) during hallucination epochs compared to baseline epochs, which they attribute to an attentional shift towards the visual percept, followed by the initiation of motor activity to press the button to report the hallucination. The authors refer to this as a general alpha-inducing effect of the steady state under Ganzfeld stimulation. Alpha synchronization has been proposed to be an indicator of an “idle state” related to “mental inactivity” (Adrian & Matthews, 1934; Pfurtscheller et al., 1996). In contrast to this idle state assumption, the inhibition hypothesis argues that alpha synchronization reflects the active inhibition of cortical areas that process sensory information during internally-directed attention (Klimesch et al., 1999; Ray & Cole, 1985). Pütz et al. (2006) argued that increased alpha activity under Ganzfeld stimulation points more towards the inhibition hypothesis than the cortical idle state assumption because Ganzfeld-induced mental state reflects more internally directed attention and spontaneous appearance of imagery (Cooper et al., 2003). The authors further argued that cortical areas involved in sensory information processing require inhibition in order for internally directed attention to occur.

Our investigation into the neural and behavioral correlates preceding Ganzfeld-induced hallucinations and decays builds upon prior work exploring perceptual phenomena such as visual phantoms and other forms of perceptual filling-in, where subjective experience can be generated or altered in the absence of complex sensory stimulation (Levinson & Baillet, 2022; Meng et al., 2005). Research on these illusions demonstrates that conscious perception can be associated with underlying neural activity and suggests that specific changes in neural states and even subtle behaviors like microsaccades may precede the subjective experience of perceptual changes or illusory content. These findings align with theoretical frameworks, such as predictive processing, which proposes that conscious perception is influenced by the brain's internal models and predictions, particularly when external sensory input is uncertain or weak, ambiguous, or uniform, a key feature of Ganzfeld stimulation. Based on this literature, which highlights the importance of examining neural and behavioral dynamics preceding altered perceptual states, we focused our analysis on the periods immediately preceding reported hallucinations and decays in the Ganzfeld.

In the present study, we aimed to investigate the potential of Ganzfeld stimulation to induce altered states of consciousness using a Ganzfeld space as opposed to the Ganzfeld goggles that were used in previous Ganzfeld studies in the field of consciousness. Earlier reported results from this project indicated that the Ganzfeld space offers a more immersive experience with a wider set of reported percepts and sensations such as bodily sensations of imbalance and dizziness (Pistolas & Wagemans, 2025). Moreover, we set out to delve deeper into the subjective experience by conducting interviews to capture the qualitative experience and relate it to our quantitative measures. We employed a multi-method approach to offer more insight into both the behavioral and neural components involved via co-registration of EEG and eye-tracking. One prominent motivation for undertaking this study was the publicly widespread assumptions surrounding the Ganzfeld effect, and particularly Ganzfeld goggles, currently being sold on various web shops with the premise that Ganzfeld stimulation can “relax your mind”. Clear empirical evidence on potential mental well-being effects resulting from Ganzfeld stimulation is, however, lacking. Before clinical implications can be suggested, there is important groundwork to be done pertaining to the impact Ganzfeld stimulation can have on the human mind. This study aimed to empirically investigate the Ganzfeld experience using a multi-method approach combining subjective self-reports, eye-tracking, and EEG. Building on broader discussions on the Ganzfeld's capacity to induce altered states of consciousness and its associations with relaxation, the present research focused on three core objectives: (1) to characterize the subjective perceptual, emotional, and phenomenal qualities reported by participants during the Ganzfeld; (2) to examine behavioral dynamics by analyzing eye movement patterns across distinct perceptual epochs (baseline, hallucination, decay); and (3) to identify neural correlates of these experiential states by comparing EEG spectral power, specifically alpha and theta band activity, across epochs. Additionally, we conducted an exploratory analysis linking individual differences in peak alpha frequency to subjective time perception. While public interest in the Ganzfeld’s potential for relaxation forms part of the broader context, the primary goal of this study was to examine the dynamic relationship between subjective experience and physiological markers throughout the Ganzfeld experience.

The goal of this paper is to offer more insight into the phenomenal and emotional experience, in addition to the potential alterations in consciousness elicited by Ganzfeld stimulation and the behavioral (eye-tracking) and neural (EEG) components involved. Although this study is rather exploratory, the eye-tracking and EEG data will mainly be analyzed with respect to the temporal dynamics of the subjective perceptual experience, self-reported using the Arduino dial. The extracted periods of baseline, hallucination and (pre-)decays will be compared in terms of the gaze dispersion and EEG signal. We focused on alpha-band activity in the EEG based on prior research linking alpha oscillations with a range of internal states, including relaxed wakefulness (Baskaran et al., 2012; Bazanova & Vernon, 2014; Klimesch, 1999; Palva & Palva, 2007). However, we recognize that alpha activity is not uniquely indicative of relaxation and may reflect a variety of neural and cognitive processes. Based on past research (Pütz et al., 2006), we hypothesize that hallucination reports will be preceded by alpha accelerations. We will use a peak-frequency time course method to find the frequency at which the power spectral density is maximal instead of the ratio between low and high alpha frequency because of its sensitivity to individual differences (Katyal et al., 2019), which are known to be substantial regarding alpha activity (Klimesch, 1997; Posthuma et al., 2001). The peak-frequency method is a more principled approach than the ratio, because it uses the entire alpha range continuously rather than dividing it into upper and lower halves. Furthermore, we expect increased alpha activity related to decays of color and brightness as reported by Cohen (1960). Theta waves were also investigated as a more exploratory analysis due to their connection with imagery (Bhattacharya, 2009).

## METHODS

### Study Design and Participants

**Figure 1.**
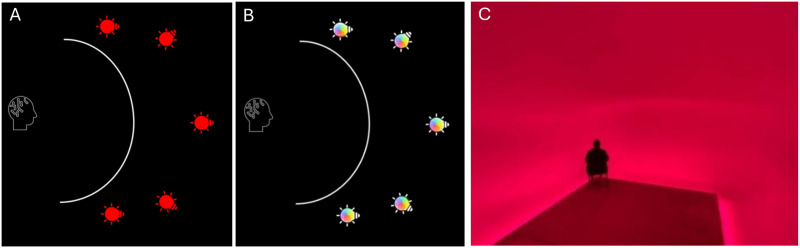
(A) Experiment 1: Observer in front of translucent hemisphere, lit from the outside using 5 lights that remain red. (B) Experiment 2: Observer in front of translucent hemisphere, lit from the outside using 5 lights that gradually change color. (C) Ganzfeld light installation by Jaap van den Elzen (changing colors). Figures licensed under CC BY 4.0 by the authors. Retrieved from https://doi.org/10.6084/m9.figshare.27890142.v1, https://doi.org/10.6084/m9.figshare.27890460.v1, https://doi.org/10.6084/m9.figshare.27890529.v1.

In the first experiment, 28 participants viewed an in-lab red Ganzfeld (GF) stimulus. In a second experiment, 45 participants were exposed to the in-lab GF with varying colors. The laboratory set-up used for Experiments 1 and 2 consisted of a translucent curved screen with 5 light sources suspended behind it as depicted in Figure 1 A and B.

The color transitions in Experiment 2 were programmed using a custom Python (https://www.python.org/) script by gradually mixing the colors red, orange, red, purple and blue (in this sequence). Each color was projected for 5 minutes with 15-second color transitions in between. More details about the specific mixing of the colors can be found in our Python script used to automate the light experience shared on our OSF page.

In a third experiment, 67 participants viewed a Ganzfeld installation artwork ‘Enter the Light’ (see Figure 1C) by Dutch artist Jaap van den Elzen (Van den Elzen, 2022).

Audio was a between-subjects factor in all three experiments with a no-noise, a white-noise and a brown-noise condition. The noise files were generated using Audacity 3.2.1 (https://audacityteam.org/). The audio was delivered using in-ear headphones (Sony LinkBuds S). Participants were able to adjust the volume to a loudness that is comfortable for them for 25 minutes. Ethical approval for this study was granted (SMEC G-2022-5469-R2(AMD)). Participants were recruited through our university experiment sign-up tool, word of mouth and advertisements on social media for Experiments 1 (25 female, 1 male, 1 preferred not to disclose, *M*_age_ = 23.46, *SD* = 8.82) and 2 (27 female, 18 male, 1 non-binary, *M*_age_ = 23.46, *SD* = 8.82), and through word of mouth and advertisements on social media for Experiment 3 (42 female, 25 male, *M*_age_ = 37.34, *SD* = 14.27). A non-Ganzfeld resting-state condition was not included in this study, because previous research that included baseline or resting-state conditions has consistently demonstrated distinctive effects associated with Ganzfeld stimulation that differentiate it from simple rest (Glicksohn et al., 2017; Schmidt et al., 2020). Thus, the observed phenomena in the present study, such as alterations in time perception, are interpreted as characteristic of the Ganzfeld-induced altered state.

### Measures

Participants were exposed to a GF for 25 minutes while EEG was recorded using the 24-channel Smarting Mobi (https://mbraintrain.com/smarting-wireless-eeg/) and eye-tracking was conducted using the Tobii Pro glasses 3 (https://www.tobii.com/products/eye-trackers/wearables/tobii-pro-glasses-3). Participants were given a box with a dial to signal the occurrence and intensity of hallucinations (range above a neutral reference point) and the occurrence of decays of color and brightness (range below the neutral point). Participants were instructed beforehand to try the dial out while looking at a live visualization of the data as they turned the dial. We instructed participants to use the dial to indicate the presence and intensity of two distinct types of perceptual experience: hallucinations, defined as emergent percepts not physically present (e.g., imagery, shapes, or sounds), and decays, defined as the fading of the color in the homogeneous visual field itself (e.g., dimming, black-out). Movement above the neutral reference point of the dial indicated hallucinations, while movement below indicated decays. This intuitive mapping served to minimize cognitive demand and preserve the immersive nature of the Ganzfeld state, which can be disrupted by overly complex tasks. The visual field remained homogeneous throughout, red in Experiment 1, and gradually changing in color in Experiments 2 and 3. Detailed analysis of the content and phenomenology of these experiences was conducted through qualitative, inductive content analysis of semi-structured interviews. This approach allowed us to preserve the integrity of the subjective experience during the session while later capturing its richness and complexity without requiring participants to make fine-grained real-time distinctions.

This dial was made using a rotary encoder, the data of which are read out using Arduino (https://www.arduino.cc/), sent out through USB and recorded using a custom Python script. The experiment script includes more details about the integration and synchronization of data streams and can be accessed through our OSF page. More specific details pertaining to the methodology can be found in earlier work that focuses on the phenomenology of the emerging and decaying visual phenomena (Pistolas & Wagemans, 2025). Epoch definitions for EEG and eye-tracking analyses differed due to the distinct nature of the signals and potential confounds. Specifically, EEG epochs were restricted to time windows without dial movement to avoid motor-related artifacts that contaminate spectral estimates, whereas eye-tracking analyses included periods with and without movement.

Following the 25-minute GF experiment session, participants were interviewed and asked to complete a questionnaire that assessed alterations in consciousness (11-ASC; Studerus et al., 2010), liking and beauty, personality and demographics. The VAS (i.e., visual analogue scale) of the 11-ASC is anchored as “No, not more than usual” on the left and as “Yes, very much more than usual” on the right. Participants were then asked to report how much they liked the light installation experience (“Did you like the light installation experience?”, 0 = “No, not at all”, 10 = “Yes, very much”) and how beautiful they found the light installation to be (“Did you find the light installation, i.e., what you experienced, beautiful?”, 0 = “No, not at all”, 10 = “Yes, very much”). The Altered States of Consciousness Rating Scale (11-ASC) was used to assess participants’ subjective experiences during the Ganzfeld exposure. This scale is specifically designed to measure deviations from an individual’s normal waking state of consciousness. A score significantly above zero indicates a perceived increase in that dimension of experience relative to the participant’s usual state. This approach follows the validated use of the scale as outlined by Dittrich et al. (2006, 2010) and Studerus et al. (2010), and is standard in both pharmacological and non-pharmacological ASC research (Schmidt et al., 2020; Schmidt & Prein, 2019). The statistical approach of testing scores against the zero anchor allows for the quantification of subjective change without the need for a separate pre-test baseline, and it enables comparisons across studies that induce ASCs using different methods, analyzed in the same way (e.g., Schmidt & Prein, 2019).

Personality was assessed using 7 International Personality Item Pool (IPIP) scales (Goldberg et al., 2006), i.e., “Artistic interests”, “Attention to emotions”, “Conscientiousness”, “Creativity”, “Curiosity”, “Imagination” and “Spirituality”. In addition, the Launay–Slade Hallucination Scale (LSHS-R) was used to assess predisposition to hallucinations (Launay & Slade, 1981). The demographical questions assessed age, gender, art interest, whether they have any experience with meditation techniques, whether they have ever tried any hallucinogenic drugs and whether they have ever been diagnosed with a neurological or psychiatric illness and what the diagnosis was. During the half-open interviews^1^, the experimenter asked participants how their experience was, what they saw, heard, smelled, felt in terms of bodily sensations, how they felt pertaining to their emotions, how the atmosphere felt, and whether they would like to share anything else about their experience. The detailed set of questions is specified in Pistolas and Wagemans (2025). The interviews took around 10 minutes per participant and were conducted in either Dutch or English for non-native Dutch speakers. Audio was recorded using a tablet.

### Qualitative Inductive Content Analysis

The interview data were analyzed using an inductive content analysis approach. In a first step, two of the research assistants listened to all interview recordings and transcribed them. Next, one of the two went over both transcriptions to ensure no information was missed and to ensure consistency in understanding the recordings. In a following step, the Dutch transcriptions were translated to English. We conducted the inductive content analysis approach to each broad interview question separately, for example visual hallucinations, emotional valence, bodily sensations, atmosphere, etc. The inductive content analysis entailed three main steps. First, a phase of familiarization during which two of the research assistants read all transcribed responses to identify recurring themes and develop categories. Second, the coding phase entailed coding all responses into relevant categories, extracting example responses for each category, and defining the categories. In the final phase of synthesis, we identified related categories and merged them into broader categories. An interrater approach was applied to ensure reliability. As such, the three phases were completed by two of the research assistants, after which they discussed inconsistencies, followed by a final check of one of the authors (who is one of the two research assistants) to merge the results based on the discussed criteria to ensure consistency.

### Eye-Tracking

Some participants’ eye-tracking data was lost due to connection issues between the Python script and the Tobii Wi-Fi connection, resulting in sample sizes of 27 for Experiment 1, 45 for Experiment 2, and 63 for Experiment 3. The eye-tracking data were preprocessed using a custom pipeline specifically created for this dataset, because the pipelines offered by the Tobii Pro Lab software captured our data insufficiently due to the following obstacles. Certain parts of the raw gaze signal did not correspond to a succession of saccades and fixations after applying the software’s pipelines. The nature of the experiment could elicit the experience of dynamic (pseudo-)hallucinations with slower glissade eye movements as a result. In addition, we expected eye movements with a low amplitude because of the lack of images with focal points in our experiment setting. The potential presence of eye movements with a small amplitude and velocity, or even of smooth glissades, complicated a clear classification of fixations and saccades in our dataset, particularly considering that definitions of these concepts vary significantly within the eye movement community (Hessels et al., 2018). For these reasons, we decided to preprocess and quantify the amount of movement from the raw gaze samples. The initial step entailed extracting the raw gaze samples using Tobii Pro Lab (https://www.tobii.com/products/software/behavior-research-software/tobii-pro-lab). Beyond this step, our whole pipeline was implemented in R (version 4.3.1; R Core Team, 2024). This includes calculating each eye’s direction on the *x* and *y* axes, compared to the *z* axis (in degrees). Next, each eye and direction axes were preprocessed separately using a low-pass Butterworth filter of second order with a cut-off value of 35 Hz (following Parisot et al., 2021). A similar low-pass filter was applied on the pupil area of each eye with a cut-off value of 4 Hz.

Subsequently, we filtered the gaze samples occurring within an interval starting 100 ms before a blink and ending 100 ms after a blink. The blink periods were extended to exclude gaze samples for which the eye direction could be contaminated by blink-related pupil size variations (Choe et al., 2016). Our blink detection algorithm was based on Choe et al.’s screen-based experiments, albeit with more liberal parameters due to the lower quality of the Tobii pro glasses 3 we used compared to Choe et al.’s use of the Eyelink 1000. We ran a moving window of 100 ms with 50 ms steps over each eye direction coordinate separately. The full moving window was flagged as blink if at least 70% contained missing values, or if the maximum pupil area change over the window was unrealistically high.

In order to detect head movements, we relied primarily on the gaze coordinates displacement over a time window (in degrees) as well as the gyroscope sensor. The latter measures the rotational velocity (in degrees/s) around the 3 axes (*x* or pitch, *y* or yaw, *z* or roll). We detected head movement by thresholding the gyroscope and eye-movement signals. We interpolated the gyroscope data using a monotonic Hermite spline to modify the timestamps to align their timestamps with the timestamps of the eye movement data. Then, we smoothed the data using a low-pass Butterworth filter of second-order with a cut-off frequency of 10 Hz. The presence of head movements is detected by comparing the gyroscope velocity values with a threshold. We implemented two procedures. The first one compared the absolute velocity of each axis to the threshold. Movement is detected at a data point if at least one of the axes reaches the threshold. The second one combined the three axes by summing the absolute velocity values of each axis preceding the comparison with the threshold. Next, we ran a sliding window over the data, with a width of 200 ms and a step of 100 ms. This window width ensures that the window cannot include more than a saccade. When at least one sample of the window was flagged as head movement (above gyroscope threshold), we used the eye movements as an additional source of information to evaluate how much the head moved within the window. We estimated the maximal range of each eye coordinate on the *X* and *Y* axes (maximum coordinate minus minimum coordinate within the window). If the maximum range across the four signals (*X* and *Y* coordinates for each eye) exceeded the eye-movement threshold, all the samples within the window were flagged as head movements. This avoided removing minimal head movements. The thresholds were chosen based on visual examination of a test video recording where one of the authors produces head movements of different amplitudes around each axis while looking at a fixation cross. For the gyroscope data, we used a threshold of 4°/s and for the eye-movement data, we used a threshold on the eye direction range of 8° over the 200 ms sliding window.

After filtering out the blinks, we centered each eye direction coordinates on each axis around their median (estimated over the entire recording). Then, we filtered out the head movements. Before any analysis, we excluded participants whose recording had more than 60% of the samples filtered out.

We estimated the eye-movement summary variables per epoch of interest. These epochs entailed four types: baseline, hallucination, decay, and pre-decay (containing the time period two seconds preceding the onset of a decay), and were determined based on the continuous Arduino dial signal, through which participants continuously reported their subjective experience of these phenomena. A more detailed description of our algorithm extracting these epochs of interest can be found in Pistolas and Wagemans (2025). Gaze direction variation on each eye and each axis was estimated by calculating the standard deviation of the blink and head movement-free gaze samples (centered coordinates).

To test the differences in eye movement variations (*SD* of gaze direction coordinates), between epochs, we ran a linear mixed model with participants as a random variable, using the lme4 R package. The epoch variable is recoded as three dummy variables with baseline as the comparison level, and entered as fixed effect. Additionally, the model accounts for the intercept variance across participants (random effect). Following this statistical modelling step, we test pairwise comparisons with Tukey correction for multiple comparisons using the emmeans R package. P-values were estimated using the Kenward-Roger approximation for the degrees of freedom.

### EEG

#### Preprocessing.

EEG was recorded using the Easycap built-in 24-electrode cap according to the 10–20 system with a CMS and a DRL electrode (https://www.easycap.de/). The recordings were conducted with a sampling frequency of 250 Hz in all channels. Some participants’ EEG data with event information from the Arduino dial was incorrectly saved due to technical difficulties, resulting in sample sizes of 26 for Experiment 1, 43 for Experiment 2, and 52 for Experiment 3. Preprocessing steps were applied in Matlab (R2024b) using the EEGLAB toolbox (Delorme & Makeig, 2004). A high-pass filter with a cut-off at 0.5 Hz was applied as well as two notch filters, one between 62 and 63 Hz to account for embedded high frequency noise at 62.5 Hz that resulted from measuring impedance values of electrodes during recording, and one between 49 and 51 Hz to account for line noise. The data was re-referenced to the average. Independent component analysis was applied to flag and remove components with a high percentage of artifacts, using 90% as a general criterion and further removing components with a high artifact percentage when the power spectrum is clearly contaminated with motor activity following Delorme et al. (2007) and Cohen (2014). On average, we removed 3.32 components in Experiment 1, 4.15 components in Experiment 2, and 5.18 components in Experiment 3. In case of channel removal due to flatlining, spherical interpolation was applied to recover the channel’s activity.

Two approaches of epoch extractions were applied for the two main analyses we intended to conduct. For the spectral analysis between epochs of interest, i.e., baseline, hallucinations and decays, in *α*, and *θ* band frequency, we extracted 10-seconds epochs for hallucination epochs (starting 20 seconds and ending 10 seconds before the onset of a hallucination), 4-seconds epochs for decay epochs (starting 5 seconds before and ending 1 second before the onset of a decay), and 6-seconds epochs for baseline epochs (starting at the onset of a baseline and ending 6 seconds later). The choice for these specific timepoints are based on previous research that found maximal alpha accelerations to occur 20 to 10 seconds before the start of hallucinations (Pütz et al., 2006). Moreover, some studies showed that decays often occur very suddenly and only last briefly. Our own findings pertaining to the temporal dynamics of the perceived visual phenomena that we discuss elsewhere (Pistolas & Wagemans, 2025), relay that hallucinations last approximately 19 seconds and decays around 11 seconds. Because there is a wide range around these averages, especially due to a large number of shorter epochs of interest, we decided to use somewhat wider time ranges. We included only negative values (i.e., the time-period before the onset of the perceived experiences of decays and hallucinations) because of two reasons: (1) one can only report the perception of an experience when it is already ongoing; (2) to avoid the contamination of the signal with motor activity due to turning the dial. Epochs that overlapped with other events were manually removed by visual inspection of the data.

The second approach pertained to the investigation of time course analysis of the peak alpha frequency related to hallucinations based on a previous study that found maximum alpha accelerations to occur 20 to 10 seconds before reports of hallucinations (Pütz et al., 2006). For this analysis, we extracted epochs starting 25 seconds and ending 5 seconds before the onset of a hallucination, based on the previously found accelerations occurring 20 to 10 seconds before hallucination reports.

#### Spectral Analysis.

Artifact-free EEG epochs were FFT-transformed to obtain frequency spectra. To account for differences in epoch duration (e.g., hallucination: 10 s; decay: 4 s), power spectra were normalized by dividing by the number of time points in each epoch, expressing power as spectral density power per unit time per frequency bin and reducing potential temporal bias (Cohen, 2014; Oostenveld et al., 2011, p. 168). This approach, involving a single Fourier transform over each continuous epoch, was chosen specifically to preserve the temporal specificity to these brief, self-reported states. While windowed methods (e.g., Welch's) prioritize spectral stability at the cost of temporal resolution, we opted for a method that avoids smoothing out subtle, transient spectral dynamics that are crucial for capturing rapid fluctuations linked to dynamically evolving experiential states with both individual and epoch-to-epoch variability (Cohen, 2014; Herrmann et al., 2014, Chapter on Spectral Analysis; Tallon-Baudry & Bertrand, 1999). The boundaries of the frequency bands of interest were 8–12 Hz (*α*) and 3–8 Hz (*θ*). We did not apply 1/f detrending for the power spectral density analyses as our focus was on alpha and theta band power related to perceptual phenomena. The relatively short and variable epoch durations, particularly for decay, limited the reliability of aperiodic component estimation using current parametrization methods (Donoghue et al., 2020). Moreover, given that 1/f characteristics may themselves vary meaningfully across different perceptual states, removing this component could obscure phenomenologically relevant differences between epochs.

#### Time Course Analysis of Alpha Frequency.

To investigate the dynamic changes in peak alpha frequency, indicative of “alpha accelerations,” preceding hallucination onset, we performed a sliding window analysis of individual alpha frequency within the hallucination epochs. For each participant, these epochs contained a 20-second interval, specifically from −25 seconds to −5 seconds relative to the reported hallucination onset, across Experiments 1, 2, and 3. Each 20-second epoch was segmented into overlapping windows. A 2-second window duration was selected to provide sufficient data for reliable spectral estimation, and we employed a 1-second overlap. For each sliding window, the Fast Fourier Transform (FFT) was computed for all EEG channels, averaged across all epochs for that specific participant and window. This yielded a power spectrum for each time window. We subtracted a linear trend from the power spectrum in log-log space. This detrending was performed specifically within the 0.5–5.5 Hz frequency range, following the method described by Katyal et al. (2019), and applied independently to the spectrum of each time window. The detrended power spectrum for each window was then smoothed using a moving average filter with a span of 7 frequency points, resulting in smoothing over approximately 3.4 Hz (±1.7 Hz on either side of the central frequency) in the frequency domain. For each smoothed spectrum obtained from a sliding window, the frequency (in Hz) with the maximum power within the 7–13 Hz alpha band was identified as the individual alpha frequency for that specific time segment. This process generated a time series of individual alpha frequency values for each participant across the entire −25 to −5 second hallucination interval. From these individual alpha frequency time series, we derived two metrics to explore alpha acceleration. The maximum individual alpha frequencies (in Hz) achieved by each participant at any point within the pre-hallucination epoch represents their highest observed alpha frequency, indicative of the “fastest alpha”. The time point (in seconds relative to hallucination onset) at which each participant's individual alpha frequency reached this maximum determines the temporal dynamics of individual alpha acceleration. The median and standard error of the mean (SEM) were then calculated for these two metrics across all participants for each experiment to determine their central tendencies. This epoch-based sliding window approach, focusing on the dynamic shifts in peak alpha frequency, quantifies “accelerated alpha” as a change in the dominant alpha frequency. This allows us to pinpoint when and to what extent alpha frequency accelerates in relation to the subjective experience of hallucinations, providing a comparison with prior literature on alpha accelerations (e.g., Pütz et al., 2006).

## RESULTS

### G-ASC

The Altered States of Consciousness Rating Scale (Studerus et al., 2010) can be analyzed by computing global scores or factor scores. The global scores of Experiments 1 (*Mean* = 23.51, *SD* = 12.60), 2 (*Mean* = 27.49, *SD* = 15.15), and 3 (*Mean* = 26.87 *SD* = 14.07) showed significant deviations from zero with *t* = 9.87, *df* = 27*, p* = 1.86e-10 for Experiment 1, *t* = 12.17, *df* = 44, *p* = 1.11e-15 for Experiment 2, and *t* = 15.63, *df* = 66, *p* < 2.2e-16 for Experiment 3. Bonferroni corrections rendered *p* = 5.58e-10 for Experiment 1, *p* = 3.32e-15 for Experiment 2, and *p* = 6.600e-16 for Experiment 3. Furthermore, the non-significant differences we found between experiments (*Chi-Squared* = 1.01, *p* = 0.61) indicates the similarity in induced alterations in consciousness across all three experiments.

### 11-ASC

**Figure 2.**
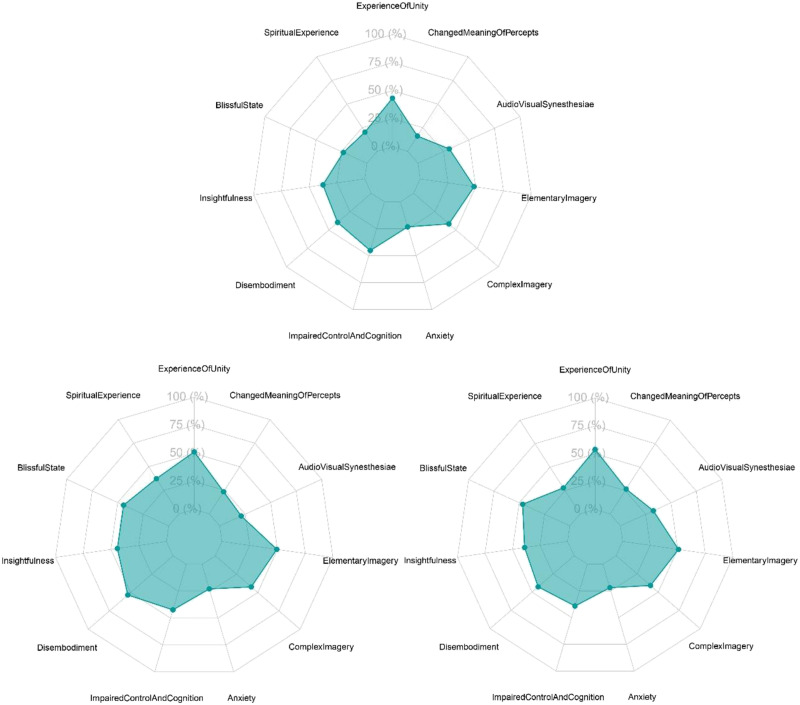
Radar chart containing average scores per dimension of Experiment 1(top), 2 (bottom left) and 3 (bottom right). Figure licensed under CC BY 4.0 by the authors. Retrieved from https://doi.org/10.6084/m9.figshare.28451426.v1.

Exploring the factors separately rendered similar results. All factors exhibited significant deviations from zero *p* < .01 for all three experiments (see Supplementary Material Tables 1, 2, and 3 containing all statistics). Figure 2 shows radar charts containing the average score per dimension for Experiments 1, 2, and 3.

### Qualitative Inductive Content Analysis

The qualitative inductive content analysis yielded a large number of categories. For reasons of brevity, we will limit our focus to the relevant themes for this paper, including time perception, depth perception, bodily sensations, and experienced feelings and emotions. Some of these results have been reported briefly in Pistolas and Wagemans (2025), here we present more details to relate the experiential level of the Ganzfeld to other measures such as eye movements and EEG activity.

#### Time Perception.

Table 1 contains the categories pertaining to distorted time perception, showing that 37.31% of the total sample reported the Ganzfeld to have felt shorter than the actual 25 minutes it lasted. More details were previously reported Pistolas and Wagemans (2025), here we relay the relevant results pertaining to alterations in self- or time-awareness. Notably, some participants (14.93%) relayed the difficulty in reporting how their time perception felt and mentioned conflicting feelings around the estimation of time and often concluded that they lacked awareness of time in the Ganzfeld. In total, across all three experiments, only four participants reported time felt longer. The remaining participants were either rather indecisive, did not give a clear answer or clearly stated that they could not answer this question.

**Table 1.** Qualitative reports regarding time perception

Category	Description	Example responses	Experiment	*N* per experiment	% per experiment	*N* total	% total
Shorter than 25 min.	The session felt shorter than 25 minutes.	no time awareness, seemed definitely shorter than 25 mins, normally when I teach I have to be very aware of time and now it was very weird to not have any awareness of time, time: went by quickly (sad it was already over) but also felt endless, time went by fast (like a meditation), did not expect to 25 minutes be over already (disorientation), thought I was only in experience for 5 mins when it ended, went by very fast, no time awareness, bad awareness of time: thought it was only 10–15 mins when it ended	1	5	18.52	50	37.31%
			2	16	39.02%		
			3	29	43.94%		
Lack of time awareness	Reports of a lack of time awareness during the experience, with instances of not knowing how long they had been there	seemed like longest 25 mins of my life but also felt like going by very quickly (but still thinking how long this is going to last), in beginning it was long and then you started and think that it is only 5 mins but then the experiment is over, I think I skipped for sure 10 mins, at certain moment I thought it was taking a long time but then when it was over it felt like a short time (it did not feel like 25 mins), really mixed, no time awareness, during experience I had no idea how far I was already, no idea how long sitting there, difficult to estimate, certain moment I was thinking how long this was still going to last but when it was over I kind of expected it	1	7	25.93%	20	14.93%
			2	3	7.32%		
			3	10	15.15%		

Figure licensed under CC BY 4.0 by the authors. Retrieved from https://doi.org/10.6084/m9.figshare.28451441.v1.

#### Depth Perception.

Table 2 shows the responses related to the distorted perception of depth in the Ganzfeld in our experiments. Besides the notable percentage of participants reporting distortions of depth, the difference between colors was also remarkable. 28.79% of participants in Experiment 3 reported that the red Ganzfeld seemed closer, and 36.36% of participants in Experiment 3 reported that the blue Ganzfeld appeared further. Similar to the time perception data, remaining participants who reported mixed, indecisive feelings were not coded into these categories. In the case of distorted depth perception, some participants had difficulty understanding what this meant. In those cases where we sensed uncertainty even after providing more explanation, we did not press for a clear answer.

**Table 2.** Qualitative reports regarding depth perception

Category	Description	Example responses	Experiment	*N* per experiment	% per experiment	*N* total	% total
Red closer	The experience of red being closer, embracing	No sense of depth but sometimes I had the feeling figures coming at me (at red-intense orange, moving images getting bigger and smaller looked like coming closer (at red, coming at me), red was bulbous: more coming at me, red: weird, free but still embraced , red was minus infinity, red: coming at you but also going away from you , certain facets of red were warmer (really felt warmth of radiation), red gave pulling out of me feeling	3	19	28.79%	–	–
Blue further	The observation of blue being more spacious, wider, further etc.	Blue was wider (in sea), more aware of the wall (but disappeared fully at blue) , losing grip on reality, felt like being under the sea at blue (more depth than normal), blue: feeling of space, infinite, pretty, feeling free, flying, blue more infinite than red: blue was plus infinity, blue gave feeling of opening up myself and diving in: I open myself to it, ocean, like how deep sea diver would feel (noise helped with this)	3	24	36.36%	–	–
Loss of depth perception	The experience of distortions in depth perception	Sometimes aware of wall but also feeling like being in a sea of colored light/endless room, looking into infinity, sometimes not seeing wall anymore, sometimes wall further or closer than normal, especially during blackout it came closer to me, no depth during blackout, in sea of blue (in beginning)	1	4	14.82%	80	58.40%
			2	26	63.42%		
			3	50	75.76%		

Figure licensed under CC BY 4.0 by the authors. Retrieved from https://doi.org/10.6084/m9.figshare.28451447.

#### Bodily Sensations.

Table 3 shows the categories pertaining to the experienced bodily sensations. A notable recurring experience was the perception of imbalance by 9.7% of the total sample across all three experiments. In the inductive content analysis, we identified a perceptually grounded type of cognitive processing concerning the bodily sensations. Within this perceptually grounded cognitive processing type, we made the distinction between externally directed and inward directed experiences. Moreover, we noticed that some reports of bodily sensations were anchored to the external environment, meaning that they emerged in relation to the external stimulation. For example, some participants specifically mentioned dizziness in relation to the loss of depth and the sensation of an endless space. These kinds of reports in which the experienced bodily sensations were related to the external stimulation were coded into the category ‘Externally directed’ and counted 34.85% of the total sample across all three experiments. In contrast, other reported bodily sensations were directed inward, for example heartbeat, temperature, etc. These were coded into the category ‘Inward directed’ and counted 34.85% of the total sample.

**Table 3.** Qualitative reports regarding bodily sensations

Category	Description	Example responses	Experiment	*N* per experiment	% per experiment	*N* total	% total
Imbalance	The sensation of losing balance and related sensations.	Light nausea, lost balance, dizzy, like color coming at you and feeling of going backwards, disorienting (bit nauseous): had to focus again and blinked a lot, lightheaded	1	2	7.41%	13	9.70%
			2	5	12.20%		
			3	6	9.09%		
Lightness, Floating, Weight	The sensation of being pulled up, descriptions related to perception of one's weight.	Like getting sucked in something when being at 1 color for a longer time, during blackout body felt lighter and floating with tingling, felt like being pulled up at blue and red, felt like floating, drifting lightly, feeling lighter, heavy feeling, drifting, heavy feeling in neck	1	1	3.70%	26	19.40%
			2	10	24.39%		
			3	15	22.72%		
Externally directed	Bodily sensations that are externally directed meaning that they emerge in relation with the outside world. For example: dizziness due to the loss of depth and the sensation of an endless space	When I looked up I had a lighter feeling dizzy like sitting on something that was spinning around feeling about to fall off chair gave bit of anxiety that continued so I was comforting myself, felt like floating and looking cross-eyed at color changes, headache at red and pink because they were so intense, lighter feeling at blue, heavier feeling at red it was annoying and I could not get it away, feeling of being on a boat…under the sea…floating, light nausea but also not really at blue	3	23	34.85%	–	–
Inward directed	Bodily sensations that are inward directed such as heartbeat, temperature, etc.	Heartbeat, more saliva, not knowing if eyes were open or closed at red/pink, tension, relaxed, cold, ice cold, felt wind, shivering at blue , warm at warmer colors like pink, like being in desert	3	23	34.85%	–	–
Eye-related	Blinking patterns and eye-related sensations	Not able to blink with eyes like normal, lots of blinking, teary eyes, dry eyes, forgetting to blink, lots of blinking (due to losing orientation), forgetting to blink	1	13	48.15%	41	30.60%
			2	9	21.95%		
			3	19	28.79%		
Posture	Noticing one's posture, such as feeling the need to adjust the posture	Sagging through chair, shoulders sagging, I felt I lost my balance, stiff from siting still, aware of how I was sitting, leaning back as time went on	1	9	33.33%	26	19.40%
			2	8	19.51%		
			3	9	13.64%		
Tiredness	Descriptions of feeling tired or sleepy, often also related to relaxation	Almost asleep, exhaustion, dopey, sagging through chair, tired, fighting with exhaustion, had trouble keeping myself awake; this was frustrating; questioning if I fell asleep, sleepy	1	19	70.37%	44	32.84%
			2	12	29.27%		
			3	13	19.70%		
Relaxation	Descriptions of various states of relaxation, such as feeling dopey, or sagging through the chair	Calmness, relaxation, sitting comfortably, at ease, relaxed, calm	1	9	33.33%	41	30.60%
			2	15	36.59%		
			3	17	25.76%		
Tension and Shaking	Noticing tension, in general or specific body parts	Tension in cheeks and forehead, biting in cheeks a few times, cramping, shivering, stiff	1	10	37.04%	29	21.64%
			2	8	19.51%		
			3	11	16.67%		
Temperature	Sensations related to temperature, including feeling cold, having cold hands, and experiencing warmth	Warm feeling, cold, cold hands, cold…sometimes gone, sometimes more intense	1	3	11.11%	20	14.93%
			2	7	17.07%		
			3	10	15.15%		
Tingling, Vibrating	Noticing tingling feelings in specific body party	Tingling in legs, itching, tingling in back, tingling in left finger, vibrating in legs	1	2	7.41%	9	6.72%
			2	2	4.88%		
			3	5	7.58%		
Loss of Reality	Reporting Instances of losing awareness of the body or grip on reality, including feeling as if sitting inside an image and being unaware of the passage of time	Sometimes I was not sure if I was holding the dial, losing depth perception, loss of reality, not sitting in chair anymore at blue, feeling I had to keep some grip, at certain moments I was gone… I was not aware anymore I was sitting on a chair, blue gave feeling of pulling in… I open myself to it, ocean, like how deep sea diver would feel and noise helped with this, I was sitting inside the image not floating, losing feeling and control of body, possibly paired with loss of depth perception, room felt smaller	1	5	18.52%	23	17.16%
			2	7	17.07%		
			3	11	16.67%		
Unpleasantness	Mention of physical discomfort, including pain in the shoulder and back, or headaches.	Back pain, unpleasant because of sitting still because I am not good in sitting still for longer periods, uncomfortable	2	4	9.76%	–	–
			3	6	9.09%		
Need to Move	Reporting a need to move, or that it was difficult to remain seated still for an extended period	Tried to sit still, feeling of falling to the front, difficult to stay seated, itchy, wanted to run away	3	8	12.12%	–	–

Figure licensed under CC BY 4.0 by the authors. Retrieved from https://doi.org/10.6084/m9.figshare.28451456.

#### Experienced Feelings and Emotions.

The description of this peculiar experience at the level of participants’ own emotions and feelings resulted in interesting qualitative data that seemed to touch upon the alterations in self-awareness. Table 4 contains the relevant categories extracted from the data. The categories “Meditative and contemplative experiences” and “Altered awareness” are particularly interesting for the focal point of this paper, i.e., the link between Ganzfeld experiences and alterations in consciousness. In addition, we identified another type of cognitive processing that recurred in this experience, characterized by reports of transcendental feelings, less awareness of the body, a loss of grip, hypnosis, meditation, spirituality and a loss of reality. This cognitive processing type was named ‘Abstract cognition’ due to the abstract nature of these experiences, seemingly touching upon the peculiarity of the experience and the focus on the self.

**Table 4.** Qualitative reports regarding experienced feelings and emotions

Category	Description	Example responses	Experiment	*N* per experiment	% per experiment	*N* total	% total
Relaxing	Participants report calming and relaxing experiences.	Calm, relaxing, calming, calming/zen-like, calm modus, calm, at ease, nice to experience something like this, like I was laying outside, rushed feeling but also calming experience, serene	1	13	48.15%	77	57.46%
			2	19	46.34%		
			3	45	68.18%		
Intense	The overall experience, but also often specific experiences were described to be intense.	Very intense experience, sadness: very intense, red, pink and blue were intense, blackouts were intense, pretty intense, intriguing	1	8	29.63%	26	19.40%
			2	4	9.76%		
			3	14	21.21%		
Meditative and Contemplative Experiences	The responses highlight the introspective aspect of the experience, where individuals feel a sense of meditation and reflection.	Contemplative: turned towards yourself, zen, calming , Experience doing as an individual, Very closed off, nobody can bother you, dependent on you as a person what you are going to see, like a meditation, contemplative, meditative, transcendental, it is something you go to and you discover what goes through your eye balls and brain, same feeling like meditating	1	3	11.11%	19	14.18%
			2	4	9.76%		
			3	12	18.18%		
Altered Awareness	Mentions that conscious was altered or experiencing hallucinations (e.g., dissociation from reality and immersion into an alternate or surreal world).	Loss of grip on reality, like being in another world, turned towards yourself, daydreaming, being in trance, felt like another world, I needed time to come back, visual hallucinations, confusing, you will be daydreaming without noticing and then you start seeing things and you go with it and then you see things and then you try to focus again, but when you are with it you see movements , getting sucked into what seeing around you like when you did not have any experience, feeling of not having a grip in reality (what am I looking at?), sinking away	1	3	11.11%	30	22.39%
			2	8	19.51%		
			3	19	28.79%		
Abstract cognition	Transcendental feelings, less awareness of body, loss of grip, hypnosis, meditation, spiritual, loss of reality	Felt like being in a trance: away from everything around me, meditative, feeling of not having a grip on reality (what am I looking at?), sinking away, felt like another world, I needed time to come back, same feeling as meditating: aware but also you are not there	3	24	35.82%	–	–

Figure licensed under CC BY 4.0 by the authors. Retrieved from https://doi.org/10.6084/m9.figshare.28451465.

### Eye-Tracking

The eye-tracking data were first analyzed by examining how much the eyes exhibited movements in the different epochs of interest. We employed the standard deviation on the *x*- and *y*-axes of each eye as a measure of dispersion for a more fine-grained analysis. Across all three experiments and on both the *x*- and *y*-axes, we found significantly less eye movements during the decays and pre-decay periods (containing 2 seconds preceding the start of a decay) compared to baseline. Across all three experiments, but only on the x-axis coordinates, we additionally found significantly less eye movements during the decays and pre-decay periods compared to hallucination epochs. Overall, the eyes seem to exhibit less pronounced eye movements on the *y*-axis compared to the *x*-axis. The pairwise comparison test statistics can be found in Supplementary Materials Tables 4–15.

#### Experiment 1.

**Figure 3.**
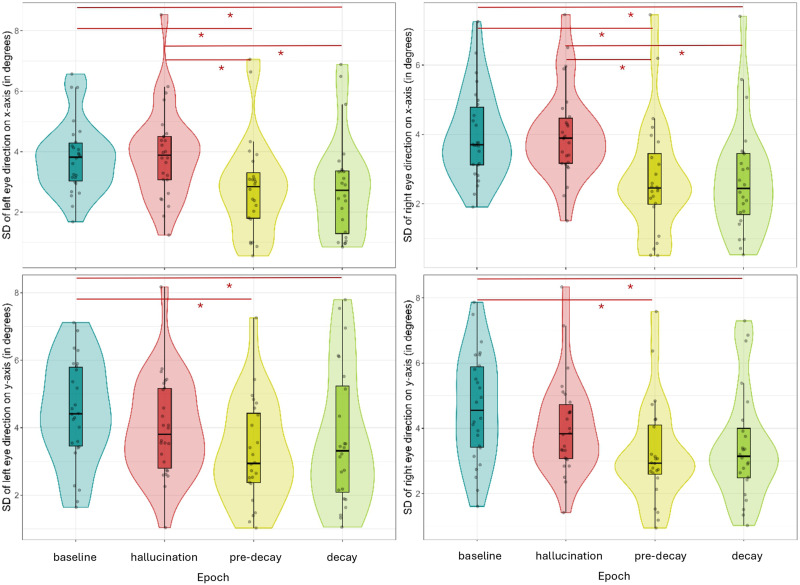
Violin plots of the standard deviation on the *x*- (top) and *y* (bottom)-axes of the left and right eyes in the different epochs of interest in Experiment 1. Figure licensed under CC BY 4.0 by the authors. Retrieved from https://doi.org/10.6084/m9.figshare.28451468.

Figure 3 (top) shows the difference in standard deviation of eye direction on the *x*-axis between epochs of interest in both eyes with the left eye on the left and the right eye on the right. Decay and pre-decay epochs contained significantly less dispersion than baseline and hallucination epochs. Figure 3 (bottom) depicts the difference in standard deviation of eye direction on the y-axis between epochs of interest in both eyes. The results here are similar to the ones of the *x*-axis but less pronounced, with only significantly less dispersion during decay and pre-decay epochs compared to baseline but not hallucination.

#### Experiment 2.

**Figure 4.**
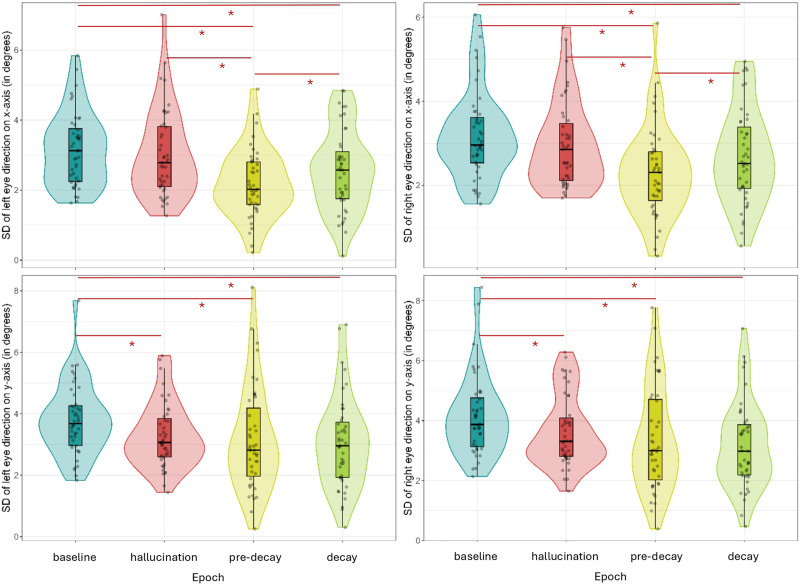
Violin plots of the standard deviation on the *x*- (top) and *y* (bottom)-axes of the left and right eyes in the different epochs of interest in Experiment 2. Figure licensed under CC BY 4.0 by the authors. Retrieved from https://doi.org/10.6084/m9.figshare.28451474.

Similar to the results in Experiment 1, we found significantly less dispersion during decay and pre-decay epochs than during baseline and hallucination epochs on the *x*-axis, depicted in Figure 4 (top). In addition, there is also a significant difference between decay and pre-decay epochs. On the *y*-axis we found significantly less dispersion during decay and pre-decay epochs compared to baseline. Furthermore, there was significantly less dispersion during hallucination epochs compared to baseline epochs, as can be seen from Figure 4 (bottom).

#### Experiment 3.

**Figure 5.**
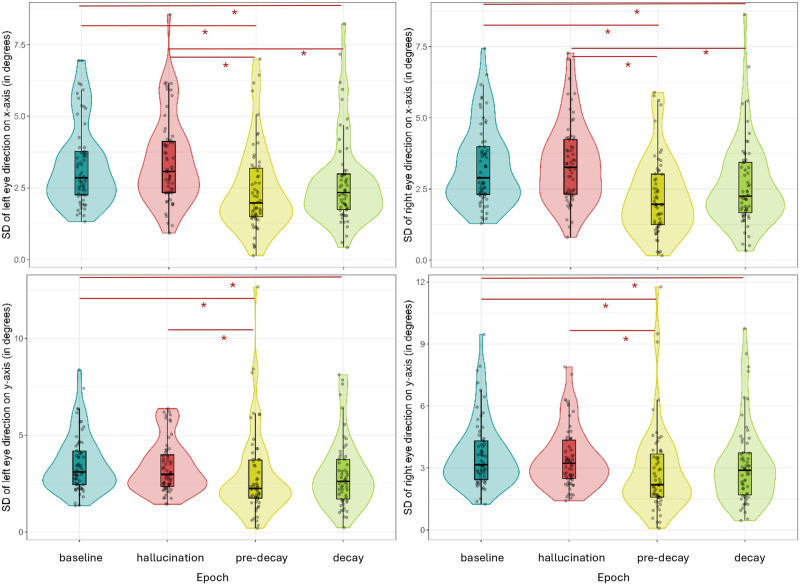
Violin plots of the standard deviation on the *x*- (top) and *y* (bottom)-axes of the left and right eyes in the different epochs of interest in Experiment 3. Figure licensed under CC BY 4.0 by the authors. Retrieved from https://doi.org/10.6084/m9.figshare.28451501.

In line with the previous two experiments, Experiment 3 again shows significantly less dispersion on the *x*- axis during decay and pre-decay epochs compared to both baseline and hallucination epochs, shown in Figure 5 (top). On the *y*-axis, we found similar results, i.e., significantly less dispersion during decay and pre-decay epochs compared to baseline epochs. Finally, the analysis on this sample also rendered a significant decrease in eye movements during pre-decay epochs compared with hallucination epochs on the y-axis, depicted in Figure 5 (bottom).

### EEG

#### Power Spectral Density.

**Figure 6.**
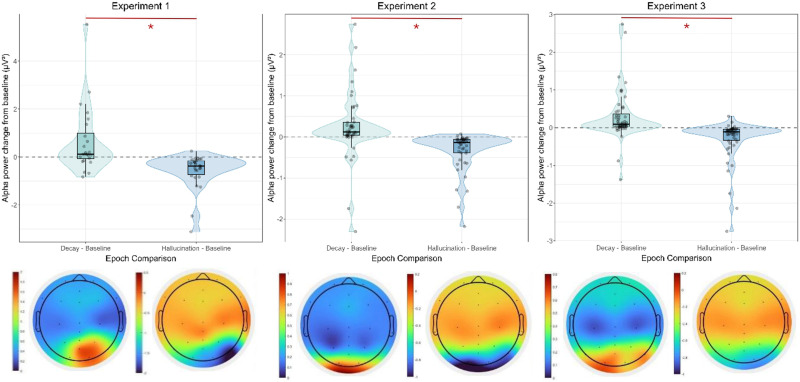
Violin plots of alpha power change from baseline in the different epochs of interest and corresponding difference topographical plots of alpha power for Experiment 1, Experiment 2, and Experiment 3, showing increased alpha power occipitally for decay epochs relative to baseline and hallucination epochs. The left plots show decay-baseline, the right plots show hallucination-baseline. Figure licensed under CC BY 4.0 by the authors. Retrieved from https://doi.org/10.6084/m9.figshare.29371379.

We examined alpha and theta band power, averaged across channels, by analyzing baseline-subtracted values across the epochs of interest (decay and hallucination, both relative to baseline). Linear mixed models (LMMs) were used, with the difference values as a fixed effect and participant as a random effect. This approach allows for direct examination of power changes from individual baseline periods. Figure 6 shows the baseline-subtracted alpha power for the epochs of interest, including the topographical contrast plots.

For Experiment 1, the LMM on baseline-subtracted alpha power showed a significant main effect of epoch. Alpha power was significantly increased during the decay epoch relative to baseline (*M* = 0.64 *μ*V^2^, *SE* = 0.22, *t*(48) = 2.86, *p* = 0.006, 95% CI [0.19, 1.09]). Alpha power was significantly decreased during the hallucination epoch relative to baseline (*M* = −0.58 *μ*V^2^, *SE* = 0.22, 95% CI [−1.02, −0.15]), as its confidence interval did not include zero. The direct comparison revealed that the increase in alpha power during decay was significantly larger than the change observed during hallucination (*b* = 1.23, *SE* = 0.31, *df* = 24.47, *t* = 3.94, *p* = 0.0006).

In Experiment 2, the LMM also revealed a significant main effect of epoch on baseline-subtracted alpha power. Alpha power was significantly increased during the decay epoch relative to baseline (*M* = 0.25 *μ*V^2^, *SE* = 0.10, *t*(83.70) = 2.41, *p* = 0.018, 95% CI [0.043, 0.45]). Alpha power was significantly decreased during the hallucination epoch relative to baseline (*M* = −0.35 *μ*V^2^, *SE* = 0.10, 95% CI [−0.56, −0.15]), as its confidence interval did not include zero. The increase in alpha power during decay was significantly larger than the change observed during hallucination (*b* = 0.60, *SE* = 0.14, *df* = 42.00, *t* = 4.26, *p* = 0.0001).

For Experiment 3, the LMM similarly demonstrated a significant main effect of epoch on baseline-subtracted alpha power. Alpha power was significantly increased during the decay epoch relative to baseline (*M* = 0.29 *μ*V^2^, *SE* = 0.085, *t*(98.80) = 3.39, *p* = 0.001, 95% CI [0.12, 0.46]). Alpha power was significantly decreased during the hallucination epoch relative to baseline (*M* = −0.31 *μ*V^2^, *SE* = 0.085, 95% CI [−0.48, −0.15]). The increase in alpha power during decay was significantly larger than the change observed during hallucination (*b* = 0.60, *SE* = 0.11, *df* = 50.00, *t* = 5.31, *p* < .001).

**Figure 7.**
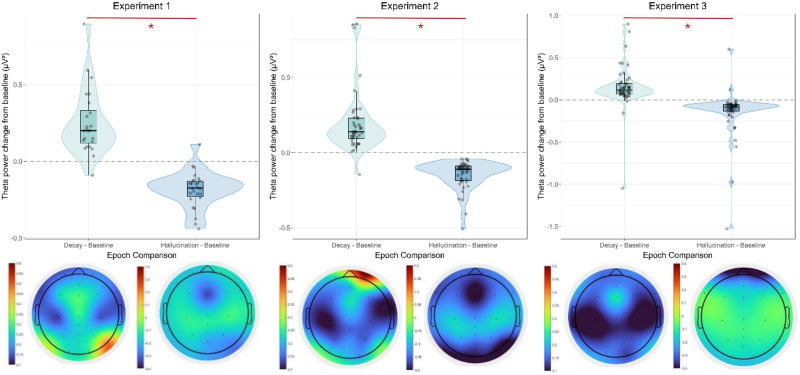
Violin plots of theta power change from baseline in the different epochs of interest and corresponding topographical plots of theta power for Experiment 1, Experiment 2, and Experiment 3, showing increased theta power midfrontally for decay epochs relative to baseline and hallucination epochs. The left plots show decay-baseline, the right plots show hallucination-baseline. Figure licensed under CC BY 4.0 by the authors. Retrieved from https://doi.org/10.6084/m9.figshare.29371382.

Regarding theta power, we found similar results. Figure 7 shows the baseline-subtracted theta power for the epochs of interest, including the topographical contrast plots.

For Experiment 1, the LMM on baseline-subtracted theta power revealed a significant main effect of epoch. Theta power was significantly increased during the decay epoch relative to baseline (*M* = 0.25 *μ*V^2^, *SE* = 0.035, *t*(48) = 7.19, *p* < .001, 95% CI [0.18, 0.32]). Theta power was significantly decreased during the hallucination epoch relative to baseline (*M* = −0.18 *μ*V^2^, *SE* = 0.034, 95% CI [−0.25, −0.12]). The direct pairwise comparison between ’Decay - Baseline' and ’Hallucination - Baseline' showed a significant difference, with theta power changes during decay being higher than during hallucination (*b* = 0.44, *SE* = 0.048, *df* = 24.47, *t* = 9.01, *p* < .001).

In Experiment 2, the LMM on baseline-subtracted theta power similarly revealed a significant main effect of epoch. Theta power was significantly increased during the decay epoch relative to baseline (*M* = 0.20 *μ*V^2^, *SE* = 0.025, *t*(84.00) = 7.89, *p* < .001, 95% CI [0.15, 0.25]). Theta power was significantly decreased during the hallucination epoch relative to baseline (*M* = −0.15 *μ*V^2^, *SE* = 0.025, 95% CI [−0.20, −0.10]). The direct pairwise comparison between ’Decay - Baseline' and ’Hallucination - Baseline' showed a significant difference, with theta power changes during decay being higher than during hallucination (*b* = 0.34, *SE* = 0.035, *df* = 42.00, *t* = 9.77, *p* < .001).

For Experiment 3, the LMM on baseline-subtracted theta power also revealed a significant main effect of epoch. Theta power was significantly increased during the decay epoch relative to baseline (*M* = 0.15 *μ*V^2^, *SE* = 0.037, *t*(87.66) = 4.08, *p* < .001, 95% CI [0.078, 0.23]). Theta power was significantly decreased during the hallucination epoch relative to baseline (*M* = −0.14 *μ*V^2^, *SE* = 0.037, 95% CI [−0.22, −0.071]). The direct pairwise comparison between 'Decay - Baseline' and 'Hallucination - Baseline' showed a significant difference, with theta power changes during decay being higher than during hallucination (*b* = 0.30, *SE* = 0.042, *df* = 50.00, *t* = 7.12, *p* < .001).

#### Time Course of Peak Alpha Frequency.

**Figure 8.**
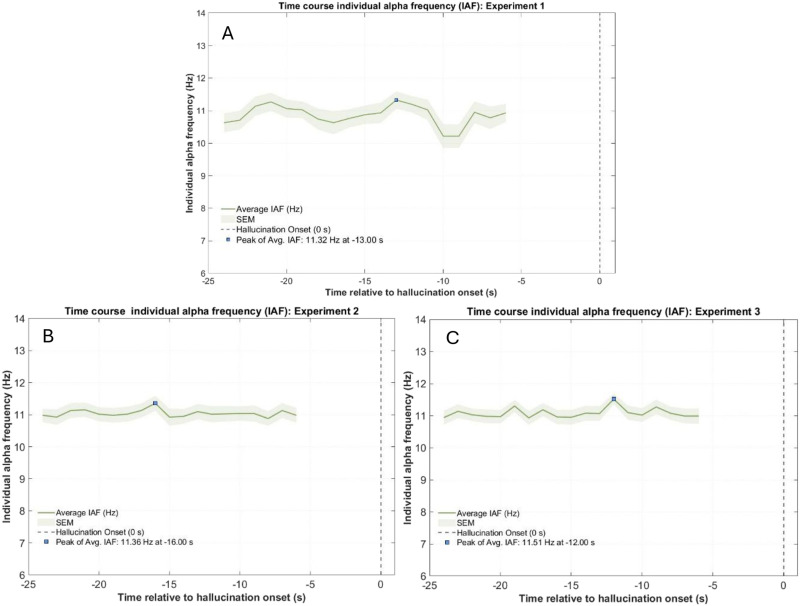
Time course of individual alpha frequency (IAF) prior to hallucination onset across experiments, illustrating the grand-averaged time course of individual alpha frequency in the 20-second epoch preceding reported hallucination onset for: (A) Experiment 1 (top panel); (B) Experiment 2 (bottom-left panel); and (C) Experiment 3 (bottom-right panel). Figure licensed under CC BY 4.0 by the authors. Retrieved from https://doi.org/10.6084/m9.figshare.29371397.

The central tendencies for the fastest alpha frequency (maximum individual alpha frequency) exhibited by participants within the hallucination epoch and its timing were similar across all three experiments with mean time of fastest alpha = −13 seconds for Experiment 1, mean time of fastest alpha = −16 seconds for Experiment 2, and mean time of fastest alpha = −12 seconds for Experiment 3. Figure 8 (panels A, B, C for Experiments 1, 2, and 3, respectively) illustrates the grand-averaged time course of individual alpha frequency for each experiment. Overall, these results align with previous findings of alpha accelerations occurring 20–10 seconds preceding hallucination onsets (Pütz et al., 2006).

In addition, we tested the relationship between time perception and peak alpha frequency using logistic regressions for all three experiments as an additional exploratory analysis and found non-significant results suggesting no relationship between peak alpha frequency and time perception (Statistical results can be found in Supplementary Materials Table 16).

## DISCUSSION

The scientific study of the Ganzfeld effect has shifted in focus over the past decades. Originating from the field of visual perception in the 1930s (Metzger, 1930), this effect was mostly studied in the context of Gestalt psychology with a prominent focus on the perceptual phenomenology and the role of adaptation in the fading of color and brightness in the Ganzfeld. Over the past three decades, the Ganzfeld effect has gained more attention from the field of consciousness (Pütz et al., 2006; Wackermann et al., 2002), because of its potential to induce altered states of consciousness (Schmidt & Prein, 2019). The Ganzfeld effect has recently also garnered more attention from the general public, partially due to the rise in popularity of Ganzfeld art installations such as the ones constructed by James Turrell, a prominent light artist from the Light and Space movement. Aside from the appeal of the aesthetic experience that can be constructed by using light and more specifically a Ganzfeld as a medium, the distribution of Ganzfeld goggles through numerous web shops has also reached individuals seeking out relaxation tactics to escape the feeling of overstimulation in today’s fast-paced world. Ganzfeld-induced altered states of consciousness have often been studied using Ganzfeld goggles, and previous research has investigated altered subjective experiences, such as time perception and altered states of consciousness, in whole-body immersive environments like Whole-Body Perceptual Deprivation chambers (Glicksohn et al., 2019). However, an extensive multi-method study integrating perceptual, phenomenal, and emotional subjective experiences with simultaneous physiological measures (such as eye-tracking and EEG linked to reported phenomena) within a Ganzfeld space has been lacking. This study aimed to fill this gap by providing a more comprehensive investigation into the qualitative and quantitative subjective experience, alongside objective behavioral and neural correlates, elicited by immersion in a Ganzfeld space.

This study aimed to fill in these blanks by investigating Ganzfeld-induced altered states of consciousness using a Ganzfeld space with thorough interviews to offer a closer look into the phenomenology and appraisal value of the subjective experience. Relating these components to behavioral and neural components using the co-registration of eye-tracking and EEG is an important addition to the current state of the literature as it provides more insight into the fluctuations of different experiences in relation to the dynamics of the eyes and brain. Altogether, we present important groundwork to study the phenomenal and emotional subjective Ganzfeld experience, including the connection with relaxing and meditative states before potential clinical implications can be suggested.

Our Ganzfeld space paradigm successfully proved to induce altered states of consciousness both at the level of global scores as well as for all separate factors. Studying altered states of consciousness can offer more insight into aberrant neural processing, underlying various mental illnesses with psychotic states (Schmidt et al., 2020). In many cases, non-pharmacological methods of induction are preferred for healthy participants due to ethical and health-related considerations. In addition, the use of a less restrictive Ganzfeld space instead of Ganzfeld goggles, may provide a more immersive and comfortable, full-body experience to study these phenomena, which may also be more similar to pharmacological paradigms at the experiential level and therefore more suitable for comparison.

The inductive content analysis rendered four themes relevant for the scope of this paper: time perception, depth perception, bodily sensations, and experienced feelings and emotions. Aside from the finding that a notable percentage of our sample (37.31%) reported an underestimation of time in our Ganzfeld sessions, previously reported in Pistolas and Wagemans (2025), the inductive content analysis further also revealed the recurring difficulty in estimating the time spent in the Ganzfeld with 14.93% of our total sample relaying they experienced a lack of time awareness in the Ganzfeld. These distortions in time perception and confusion revolving around time perception in the Ganzfeld are important to point out given how salient of an altered state of consciousness characteristic altered time perception is (Wackermann et al., 2002). In our study, a notable percentage of participants experienced difficulty reporting how their time perception felt and often concluded they lacked awareness of time in the Ganzfeld. Similar observations have been reported in previous studies using immersive altered sensory environments. Glicksohn et al. (2017), using a Whole-Body Perceptual Deprivation (WBPD) chamber, which is a form of immersive Ganzfeld, found that for a majority of their participants, rating their sense of time on a numerical scale was considered a ‘nonsensical idea, ' or they were simply unable to provide a rating. Some participants in their study also explicitly reported that time ‘disappeared' or that there was an ‘absence of time' during the session. While both our study and Glicksohn et al. (2017) report instances of time feeling subjectively different (shorter or longer/expanded) for some participants, the shared observation of difficulty in reporting or lack of time awareness highlights a key characteristic of the altered state elicited by immersive Ganzfeld environments.

Similar to the time perception component, distorted depth perception was previously reported in Pistolas and Wagemans (2025). The reason we highlight the distorted depth perception in this paper is the potential role it may play in the experienced bodily sensations such as imbalance, and the inferred distinction between inward directed and externally directed perceptually grounded cognitive processing types.

The peculiarity of the subjective Ganzfeld experience was to a great extent captured in the inductive content analysis of experienced feelings and emotions reports. More specifically, the themes that came forth out of this analysis clearly allude to the experience of altered states of consciousness and meditative states. The latter was even spontaneously relayed by some participants who mentioned: “[It was the] same feeling as meditating: aware, but also you are not there”, “[It was] like a meditation”, “contemplative”, “zen”. Based on these and more extensive descriptions, we identified another cognitive processing type that recurred: abstract cognition. All responses related to transcendental feelings, less awareness of the body”, “loss of grip”, “hypnosis”, “meditation”, “spirituality”, and “a loss of reality”, were included into the abstract cognitive processing type. Although this type of processing, characterized by an introspective focus and less awareness of the body and emerging percepts, is somewhat contrasting the perceptually grounded cognitive processing type (both inward and externally directed), some participants reported to experience both. Moreover, our findings suggest that this Ganzfeld experience can be seen as a journey on which one can experience shifts in attention between exteroceptive percepts (e.g., distorted depth), interoceptive percepts (e.g., body temperature and heartrate), and introspective thoughts (e.g., contemplation, and meditative states).

Our eye-tracking data supports previous literature in which reduced eye movements have been found to precede decays of color and brightness in the Ganzfeld (Kirkwood, 1967). The continuous assessment of the perceptual experience using the dial in our study allows for a more refined analysis of the temporal dynamics in eye movements. The extraction of pre-decay epochs containing two seconds preceding the onset of a decay, allowed for a closer look into how eye movements fluctuate before decaying experiences, during decays, during hallucinations and during baseline. Our data indicate that the pre-decay and decay epochs were characterized by a reduction in eye movements compared to baseline and hallucination epochs. In addition to replicating previously reported results concerning the reduced eye movement potentials found preceding decays, our results also suggest that the pre-decay epochs contain even less dispersion than the decay epochs. This can be inferred by visual inspection of the violin plots conveying the standard deviations of both eyes on the *x*- and *y*-axes as well as from the tables containing the statistical results of the linear mixed models in Supplementary Materials. More specifically, the results in Experiment 3 (with the largest sample size) on the *y*-axis, for which there is overall less dispersion, convey significant reductions in dispersion during pre-decay epochs compared to both hallucination and baseline epochs whereas decay periods only show significantly less dispersion compared to baseline epochs and for the left eye only.

One potential explanation could be that decays are characterized by reduced eye movements to the extent that decreased eye movements are a requirement for decays to occur as suggested by Kirkwood (1967). Given the presumed cause underlying decays of color and brightness, i.e., unstructured, invariant visual stimulation, this may be a plausible explanation. Even if the visual stimulation is unstructured and uniform, given that the eyes can move and therefore detect facial features such as the nose and eyelashes, we can infer that more movement of the eye can inhibit the perceptual effects of Ganzfeld exposure and in fact, hinder Ganzfeld exposure in essence. Perceptual filling-in helps in this regard to perceive a homogeneous visual field and to ensure that the Ganzfeld effect occurs. Some participants reported their insight into this mechanism, one person specified: “When [I was] looking at a point for too long, suddenly I did not see anything anymore, I let the blackouts come to me, [they] disappeared with eye movements and disappeared a bit with blinking.” Note that in the 1970s, scleral lenses, placed directly on the eyeball, were used to study stabilized images (Koenderink, 1972). In this type of set-up, the scleral lenses were connected to an optical system that compensated for each eye movement, resulting in stabilized retinal images. One could argue that only this kind of methodology allows for a proper Ganzfeld. One could also argue that this method might have its shortcomings related to the invasiveness of the lenses, and the “meditative” and “relaxing” properties of this kind of Ganzfeld viewing could be questioned.

A second explanation can be searched for in the direction of a lag in reporting these decaying phenomena. An individual can only report an experience once they are aware of the presence of such a perceptual experience. Moreover, our interview data does contain supporting information that participants noticed a delay in reporting experiences in general and also more specific reports of difficulties in reporting the decays on time because they were so overwhelming. One participant mentioned: “Blackouts were when I was zoning out and I think I reported them later than they happened. It was often immediately after something visual and then I would only realize after, ‘oh you’re losing everything now’, and then you get into the experience again. It was in those moments of blacking out that I felt like I was being pushed back. It wasn’t a long period, it was more a wave that started in the periphery, pulsating and when it was almost completely black, I came back to awareness. It might have been completely black for a moment, but I was too late to report it because I was only aware of it later. The lags in reports were much longer for blackouts than for hallucinations because the hallucinations were mainly visual and that was it, but the blackouts played on my entire body and everything a little bit. It was easier to get out of them than to get into a blackout.”

These kinds of reports hint at the phenomenology of mind blanking. As unlikely as it seems that the human brain would stop producing visual percepts altogether under invariant, unstructured visual information, it seems just as unlikely that the human mind would never draw a blank under these circumstances, considering the notion of mind blanking, which argues that the human mind can be deprived of reportable thoughts (Mortaheb et al., 2022).

Our EEG results concerning the time course of the peak alpha frequency replicate the findings of Pütz et al. (2006) who found that maximum alpha accelerations occurred 20 to 10 seconds before a hallucination was reported using a different approach. While Pütz et al. employed the ratio between high and low alpha as an approximation of alpha accelerations, we computed peak alpha frequency. The range Pütz et al. reported is centered around the time we found, i.e., approximately 15 seconds preceding the onset of hallucinations. Regarding the power spectral density, the linear mixed models on baseline-subtracted power values revealed consistent patterns across all three experiments. Alpha power was significantly increased during the decay epoch relative to baseline and significantly decreased during the hallucination epoch relative to baseline across all experiments. Consequently, the increase in alpha power during decay was significantly larger than the change observed during hallucination consistently across all three experiments. Overall, these results replicate the previously found decrease in alpha power for hallucination epochs and additionally indicate that alpha power was highest for the decay epochs, aligning with early Ganzfeld EEG studies (Cohen, 1960; Tepas, 1962) where decays of color and brightness coincided with significantly more alpha. Cohen has described a relationship between alpha-band activity and the absence of visual stimulation. This aligns with longstanding findings suggesting that occipital alpha activity may reflect reduced sensory input or cortical idling (Berger, 1929; Jensen & Mazaheri, 2010; Klimesch, 2012), although such interpretations remain debated and context-dependent.

A “gating function” theory has been proposed to explain increased alpha activity in inhibited brain regions and reduced activity in regions actively processing information (Toscani et al., 2010). In this case, a form of visual suppression, i.e., inhibition of the visual system to process the invariant visual stimulation (Volkmann, 1986), might explain the increased alpha power we found related to decays of color and brightness of the visual field, as found in Toscani et al. (2010) using an eyes-closed paradigm of visual suppression. The observed increase in alpha power may reflect attenuated sensory processing, consistent with theories linking alpha waves to internally directed attention and decreased responsiveness to external input (Cooper et al., 2003; Pütz et al., 2006). While such dynamics have been associated with relaxed states (Baskaran et al., 2012), we acknowledge that alpha activity can emerge under diverse cognitive and perceptual conditions.

For theta power, linear mixed models on baseline-subtracted data also revealed consistent and significant changes across epochs. Theta power was significantly increased during the decay epoch relative to baseline and significantly decreased during the hallucination epoch relative to baseline across all experiments. Furthermore, the increase in theta power during decay was significantly larger than the change observed during hallucination consistently across all three experiments.

The attenuation of sensory processing that seems to recur in the Ganzfeld experience, in addition to previous findings of decreased thalamo-cortical coupling and increased default mode network (DMN) activity (Schmidt et al., 2020), point to the central role the suppression of sensory information processing may play in the Ganzfeld experience. The earlier proposed link with mind blanking and specifically, the question as to whether there might be an overlap between the experienced decays reported in the Ganzfeld and the phenomenon of mind blanking is raised once again given the similarities in neural processing. Instructing participants to ‘think of nothing’, Kawagoe et al. (2018) found a decrease in connectivity between the DMN and the sensory areas of the cortex. Moreover, Andrillon et al. (2021) found that slow waves (delta or theta range) preceded mind blanking and mind wandering. The authors argue that the slow waves might inhibit communication between parietal and frontal areas, resulting in mind blanking. This suggested lack of connectivity and thus communication between frontal and parietal brain areas underlying mind blanking is further supported by the whole-brain deactivations found preceding mind blanking by Boulakis et al. (2023). Our findings pertaining to increased theta power related to the decay epochs compared to both hallucination and baseline epochs could be indicative of this lack of communication between brain areas.

Our observation of decreased eye movements and specific changes in alpha and theta power preceding decay experiences aligns with findings from studies on related perceptual phenomena, such as perceptual filling-in (Levinson & Baillet, 2022). Prior research on filling-in has demonstrated that a reduction in eye movements, particularly microsaccades, often precedes the subjective experience of sensory information fading or being filled in. This suggests a link between oculomotor stillness and the dissolution of stable perception, consistent with our finding of decreased eye movements before Ganzfeld decays. Furthermore, our neural findings of increased theta activity and higher alpha power preceding decays can be framed within predictive processing accounts and studies of altered states. Increased alpha power is often associated with attenuated sensory processing and relaxed states, potentially reflecting a down weighting of unreliable sensory input in the uniform Ganzfeld (Hohwy & Seth, 2020). The increase in theta power preceding decays is consistent with the notion of mind blanking, a transient lapse in conscious thought (Mortaheb et al., 2022), which may occur as the brain struggles to sustain a coherent perceptual model in the absence of structured input. These results, viewed through the lens of perceptual filling-in and predictive processing, support the idea that changes in internal neural states and associated behaviors contribute to the subjective experience of perceptual alterations, such as decays, particularly when the sensory environment is uniform, leading to increased reliance on internally generated predictions or a disruption in predictive processing (Hohwy & Seth, 2020).

## CONCLUSIONS

Aiming to fill in the blanks of the multifaceted Ganzfeld pertaining to the perceptual, phenomenal, and emotional experience using a multi-method approach by combining behavioral (eye-tracking) and neural (EEG) measures, along with qualitative (interviews) and quantitative (questionnaires and rating scales) assessments, we can now draw various conclusions. A first point we raise for future research regarding Ganzfeld-induced altered states of consciousness is the suggestion to use the less restrictive Ganzfeld space instead of Ganzfeld goggles when appropriate (i.e., when the methodology allows for such a free setting) in view of the full-body experience it permits. The underestimation of time perception and uncertainty around time awareness we found, further highlight the induction of altered states of consciousness in the Ganzfeld. Distorted depth perception seemed to play a central role in the bodily sensations of imbalance prompting us to differentiate between inward-directed and externally directed perceptually grounded cognition. Aside from this cognitive processing type characterized by awareness of and a focus on the body, we identified the emergence of an abstract cognitive processing type denoted by an introspective focus with self-reported “meditative”, or “contemplative” states of “relaxation”. At the behavioral level, our eye-tracking data indicated that decays of color and brightness coincide with and are preceded by reduced eye movements. The reduction in eye movements seems even more prominent during pre-decay periods than decays. As such, a reduction in eye movements might be a necessary requirement for decays to occur. Another potential explanation could be a larger lag in reporting decays due to the overwhelming nature of these decays, sometimes followed by “coming back to awareness”. The way the subjective experience of decays has been reported points to the concept of mind blanking. The increased theta activity observed during decays may resemble patterns reported in prior studies of mind-blanking (e.g., Kawagoe et al., 2019), although we did not obtain concurrent reports to confirm such states and therefore rely on retrograde interview data. The concurrent increase in alpha power could indicate reduced sensory processing or shifts in attention, consistent with theories of neural inhibition (Klimesch et al., 2007). However, given the correlational nature of EEG data, we interpret the observed effects with caution and avoid drawing conclusions about specific internal experiences. Disentangling the perceptual decays from the apparent mental decays, or states similar to mind blanking, should be the focus of future work to study the cognitive processing and altered states of consciousness in more depth. More clarity regarding these mental states is necessary to investigate the relaxing and meditative component before potential mental well-being effects of Ganzfeld exposure can be suggested.

## Acknowledgments

We thank Christophe Bossens for his technical support, Liv Smets and Jirka Liessens for their assistance during data collection, and Liv Smets and Corinna Kühnapfel for their help with the inductive content analysis. In addition, we thank Ignace Hooge and Roy Hessels for all the help and suggestions they provided us regarding the eye-tracking data analysis. Their feedback was extremely useful and necessary to build our preprocessing and analysis pipeline.

## Funding Information

This project is supported by long-term structural funding by the Flemish government (METH/21/02) awarded to Johan Wagemans.

## Author Contributions

E.P.: Conceptualization; Data curation; Formal analysis; Investigation; Methodology; Project administration; Software; Validation; Visualization; Writing – original draft; Writing – review & editing. B.Q.: Data curation; Formal analysis; Software; Visualization; Writing – review & editing. S.K.: Conceptualization; Methodology; Supervision; Writing – review & editing. J.W.: Conceptualization; Funding acquisition; Methodology; Resources; Supervision; Writing – review & editing.

## Data Availability Statement

Data available upon request by contacting the corresponding author: eleftheria.pistolas@kuleuven.be.

## Note

^1^ Although we aimed at asking participants the same questions, we allowed for some degrees of freedom during the interview to allow for follow-up questions whenever it was not clear what participants meant and to better assess aspects that were mentioned repeatedly by several participants.

## Supplementary Material


