# Supplementary material for: The Multifaceted Ganzfeld at the Crossroad Between Visual Perception and Consciousness: Behavioral, Neural and Qualitative Aspects
Source: Open Mind (Camb). 2025 Nov 10;9:1906–38. doi: 10.1162/OPMI.a.255 (PMC12622472; doi:10.1162/OPMI.a.255)
Supplement: Supplementary file 1 [file opmi-09-1906-s001.docx]

**Supplementary Materials**

**Table 1**

*Factor scores Experiment 1*

| Factor | Mean | SD | Bonferroni-corrected p-value | t |
| --- | --- | --- | --- | --- |
| Experience of Unity | 30.32 | 14.58 | 1.92e-10 | 11.01 |
| Spiritual Experience | 14.20 | 14.13 | 1.42e-04 | 5.32 |
| Blissful State | 16.15 | 16.59 | 2.22e-04 | 5.15 |
| Insightfulness | 26.34 | 22.50 | 1.39e-05 | 6.19 |
| Disembodiment | 27.75 | 22.29 | 5.03e-06 | 6.59 |
| Impaired Control and Cognition | 31.65 | 17.93 | 6.62e-09 | 9.34 |
| Anxiety | 16.26 | 15.06 | 4.99e-05 | 5.71 |
| Complex Imagery | 29.13 | 24.55 | 1.12e-05 | 6.28 |
| Elementary Imagery | 34.01 | 22.98 | 2.23e-07 | 7.83 |
| Audio-Visual Synesthesiae | 21.50 | 20.65 | 8.54e-05 | 5.51 |
| Changed Meaning of Percepts | 11.29 | 14.05 | 2.50e-03 | 4.25 |

**Table 2**

*Factor scores Experiment 2*

| Factor | Mean | SD | Bonferroni-corrected p-value | t |
| --- | --- | --- | --- | --- |
| Experience of Unity | 35.92 | 22.06 | 4.52e-13 | 10.92 |
| Spiritual Experience | 26.12 | 18.15 | 2.16e-11 | 9.65 |
| Blissful State | 31.00 | 23.42 | 2.54e-10 | 8.88 |
| Insightfulness | 30.95 | 21.11 | 1.23e-11 | 9.83 |
| Disembodiment | 37.35 | 26.88 | 6.16e-11 | 9.32 |
| Impaired Control and Cognition | 29.60 | 17.24 | 7.88e-14 | 11.52 |
| Anxiety | 15.99 | 14.85 | 5.91e-08 | 7.22 |
| Complex Imagery | 29.62 | 20.84 | 3.11e-11 | 9.54 |
| Elementary Imagery | 34.61 | 28.39 | 2.47e-09 | 8.18 |
| Audio-Visual Synesthesiae | 14.78 | 17.05 | 6.98e-06 | 5.81 |
| Changed Meaning of Percepts | 16.47 | 17.17 | 8.54e-07 | 6.43 |

**Table 3**

*Factor scores Experiment 3*

| Factor | Mean | SD | Bonferroni-corrected p-value | t |
| --- | --- | --- | --- | --- |
| Experience of Unity | 37.78 | 19.63 | 2.42e-15 | 15.75 |
| Spiritual Experience | 19.50 | 15.40 | 1.93e-14 | 10.36 |
| Blissful State | 32.66 | 22.51 | 2.42e-15 | 11.87 |
| Insightfulness | 27.32 | 20.99 | 6.12e-15 | 10.65 |
| Disembodiment | 29.90 | 24.03 | 3.95e-14 | 10.18 |
| Impaired Control and Cognition | 27.43 | 18.41 | 2.42e-15 | 12.19 |
| Anxiety | 15.41 | 14.18 | 7.34e-12 | 8.89 |
| Complex Imagery | 28.61 | 24.88 | 8.86e-13 | 9.41 |
| Elementary Imagery | 35.51 | 24.67 | 2.42e-15 | 11.78 |
| Audio-Visual Synesthesiae | 22.79 | 22.64 | 1.09e-10 | 8.24 |
| Changed Meaning of Percepts | 18.61 | 19.84 | 1.10e-09 | 7.68 |

**Table 4**

*Gaze dispersion results on the x-axis of the left eye in Experiment 1*

| contrast | estimate | SE | df | t-ratio | p-value |
| --- | --- | --- | --- | --- | --- |
| baseline - hallucination | -0.145 | 0.263 | 71.0 | -0.439 | 0.9715 |
| baseline – pre-decay | 0.872 | 0.271 | 71.7 | 3.548 | 0.0038 |
| baseline - decay | 0.597 | 0.271 | 71.7 | 3.656 | 0.0027 |
| hallucination - predecay | 1.017 | 0.271 | 71.7 | 3.975 | 0.0009 |
| hallucination - decay | 0.742 | 0.271 | 71.7 | 4.083 | 0.0006 |
| pre-decay - decay | -0.275 | 0.274 | 71.0 | 0.106 | 0.9996 |

**Table 5**

*Gaze dispersion results on the x-axis of the right eye in Experiment 1*

| contrast | estimate | SE | df | t-ratio | p-value |
| --- | --- | --- | --- | --- | --- |
| baseline - hallucination | -0.0437 | 0.262 | 71.0 | -0.167 | 0.9983 |
| baseline – pre-decay | 1.2061 | 0.269 | 71.7 | 4.477 | 0.0002 |
| baseline - decay | 1.2721 | 0.269 | 71.7 | 4.722 | 0.0001 |
| hallucination - predecay | 1.2498 | 0.269 | 71.7 | 4.639 | 0.0001 |
| hallucination - decay | 1.3158 | 0.269 | 71.7 | 4.884 | <.0001 |
| pre-decay - decay | 0.0660 | 0.273 | 71.0 | 0.242 | 0.9950 |

**Table 6**

*Gaze dispersion results on the y-axis of the left eye in Experiment 1*

| contrast | estimate | SE | df | t-ratio | p-value |
| --- | --- | --- | --- | --- | --- |
| baseline - hallucination | 0.495 | 0.274 | 71.0 | 1.804 | 0.2800 |
| baseline – pre-decay | 1.201 | 0.282 | 71.6 | 4.253 | 0.0004 |
| baseline - decay | 0.771 | 0.282 | 71.6 | 2.730 | 0.0389 |
| hallucination - predecay | 0.706 | 0.282 | 71.6 | 2.500 | 0.0685 |
| hallucination - decay | 0.276 | 0.282 | 71.6 | 0.977 | 0.7629 |
| pre-decay - decay | -0.430 | 0.286 | 71.0 | -1.505 | 0.4397 |

**Table 7**

*Gaze dispersion results on the y-axis of the right eye in Experiment 1*

| contrast | estimate | SE | df | t-ratio | p-value |
| --- | --- | --- | --- | --- | --- |
| baseline - hallucination | 0.599 | 0.314 | 71.0 | 1.909 | 0.2336 |
| baseline – pre-decay | 1.398 | 0.323 | 71.8 | 4.334 | 0.0003 |
| baseline - decay | 1.167 | 0.323 | 71.8 | 3.616 | 0.0030 |
| hallucination - predecay | 0.799 | 0.323 | 71.8 | 2.477 | 0.0723 |
| hallucination - decay | 0.568 | 0.323 | 71.8 | 1.759 | 0.3014 |
| pre-decay - decay | -0.232 | 0.327 | 71.0 | -0.709 | 0.8933 |

**Table 8**

*Gaze dispersion results on the x-axis of the left eye in Experiment 2*

| contrast | estimate | SE | df | t-ratio | p-value |
| --- | --- | --- | --- | --- | --- |
| baseline - hallucination | 0.167 | 0.141 | 127 | 1.186 | 0.6370 |
| baseline – pre-decay | 1.012 | 0.141 | 127 | 7.189 | <.0001 |
| baseline - decay | 0.628 | 0.141 | 127 | 4.462 | 0.0001 |
| hallucination - predecay | 0.845 | 0.141 | 126 | 5.992 | <.0001 |
| hallucination - decay | 0.461 | 0.141 | 126 | 3.270 | 0.0075 |
| pre-decay - decay | -0.384 | 0.141 | 126 | -2.722 | 0.0368 |

**Table 9**

*Gaze dispersion results on the x-axis of the right eye in Experiment 2*

| contrast | estimate | SE | df | t-ratio | p-value |
| --- | --- | --- | --- | --- | --- |
| baseline - hallucination | 0.240 | 0.122 | 126 | 1.975 | 0.2031 |
| baseline – pre-decay | 0.961 | 0.122 | 126 | 7.863 | <.0001 |
| baseline - decay | 0.586 | 0.122 | 126 | 4.799 | <.0001 |
| hallucination - predecay | 0.721 | 0.122 | 125 | 5.890 | <.0001 |
| hallucination - decay | 0.346 | 0.122 | 125 | 2.830 | 0.0274 |
| pre-decay - decay | -0.374 | 0.122 | 125 | -3.076 | 0.0136 |

**Table 10**

*Gaze dispersion results on the y-axis of the left eye in Experiment 2*

| contrast | estimate | SE | df | t-ratio | p-value |
| --- | --- | --- | --- | --- | --- |
| baseline - hallucination | 0.513 | 0.171 | 127 | 2.997 | 0.0171 |
| baseline – pre-decay | 0.637 | 0.171 | 127 | 3.724 | 0.0017 |
| baseline - decay | 0.753 | 0.171 | 127 | 4.400 | 0.0001 |
| hallucination - predecay | 0.124 | 0.171 | 126 | 0.725 | 0.8870 |
| hallucination - decay | 0.240 | 0.171 | 126 | 1.400 | 0.5017 |
| pre-decay - decay | 0.116 | 0.171 | 126 | 0.675 | 0.9063 |

**Table 11**

*Gaze dispersion results on the y-axis of the right eye in Experiment 2*

| contrast | estimate | SE | df | t-ratio | p-value |
| --- | --- | --- | --- | --- | --- |
| baseline - hallucination | 0.530 | 0.176 | 126 | 3.017 | 0.0161 |
| baseline – pre-decay | 0.753 | 0.177 | 126 | 4.267 | 0.0002 |
| baseline - decay | 0.976 | 0.177 | 126 | 5.526 | <.0001 |
| hallucination - predecay | 0.223 | 0.177 | 126 | 1.263 | 0.5882 |
| hallucination - decay | 0.446 | 0.177 | 126 | 2.520 | 0.0617 |
| pre-decay - decay | 0.222 | 0.176 | 125 | 1.263 | 0.5881 |

**Table 12**

*Gaze dispersion results on the x-axis of the left eye in Experiment 3*

| contrast | estimate | SE | df | t-ratio | p-value |
| --- | --- | --- | --- | --- | --- |
| baseline - hallucination | -0.145 | 0.170 | 173 | -0.850 | 0.8302 |
| baseline – pre-decay | 0.872 | 0.173 | 175 | 5.046 | <.0001 |
| baseline - decay | 0.597 | 0.173 | 175 | 3.454 | 0.0038 |
| hallucination - predecay | 1.017 | 0.174 | 174 | 5.843 | <.0001 |
| hallucination - decay | 0.742 | 0.174 | 174 | 4.262 | 0.0002 |
| pre-decay - decay | -0.275 | 0.174 | 172 | -1.583 | 0.3912 |

**Table 13**

*Gaze dispersion results on the x-axis of the right eye in Experiment 3*

| contrast | estimate | SE | df | t-ratio | p-value |
| --- | --- | --- | --- | --- | --- |
| baseline - hallucination | -0.107 | 0.160 | 177 | -0.669 | 0.9088 |
| baseline – pre-decay | 1.067 | 0.162 | 178 | 6.584 | <.0001 |
| baseline - decay | 0.683 | 0.162 | 178 | 4.212 | 0.0002 |
| hallucination - predecay | 1.174 | 0.163 | 177 | 7.197 | <.0001 |
| hallucination - decay | 0.790 | 0.163 | 177 | 4.840 | <.0001 |
| pre-decay - decay | -0.384 | 0.164 | 176 | -2.351 | 0.0906 |

**Table 14**

*Gaze dispersion results on the y-axis of the left eye in Experiment 3*

| contrast | estimate | SE | df | t-ratio | p-value |
| --- | --- | --- | --- | --- | --- |
| baseline - hallucination | 0.0834 | 0.192 | 173 | 0.435 | 0.9723 |
| baseline – pre-decay | 0.6129 | 0.195 | 174 | 3.145 | 0.0104 |
| baseline - decay | 0.5421 | 0.195 | 174 | 2.782 | 0.0303 |
| hallucination - predecay | 0.5295 | 0.196 | 174 | 2.699 | 0.0380 |
| hallucination - decay | 0.4586 | 0.196 | 174 | 2.338 | 0.0936 |
| pre-decay - decay | -0.0709 | 0.196 | 172 | -0.362 | 0.9838 |

**Table 15**

*Gaze dispersion results on the y-axis of the right eye in Experiment 3*

| contrast | estimate | SE | df | t-ratio | p-value |
| --- | --- | --- | --- | --- | --- |
| baseline - hallucination | 0.164 | 0.201 | 177 | 0.815 | 0.8475 |
| baseline – pre-decay | 0.798 | 0.204 | 178 | 3.914 | 0.0007 |
| baseline - decay | 0.529 | 0.204 | 178 | 2.592 | 0.0502 |
| hallucination - predecay | 0.634 | 0.205 | 177 | 3.088 | 0.0124 |
| hallucination - decay | 0.364 | 0.205 | 177 | 1.775 | 0.2886 |
| pre-decay - decay | -0.270 | 0.206 | 176 | -1.310 | 0.5576 |

**Table 16**

*Statistical results of the relation between time and alpha acceleration*

| Experiment | Estimate | Standard Error | z-value | p-value |
| --- | --- | --- | --- | --- |
| 1 | -0.37 | 0.35 | -1.07 | 0.28 |
| 2 | -0.19 | 0.18 | -1.04 | 0.30 |
| 3 | 0.009 | 0.17 | 0.05 | 0.96 |


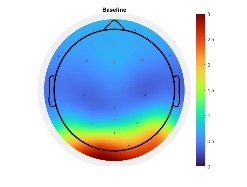

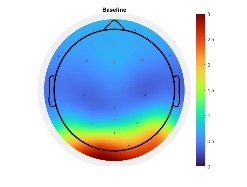

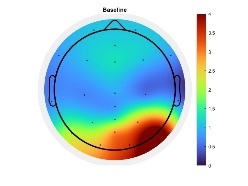

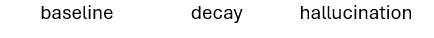

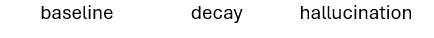

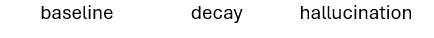

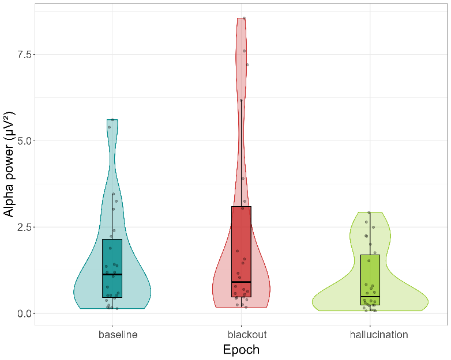

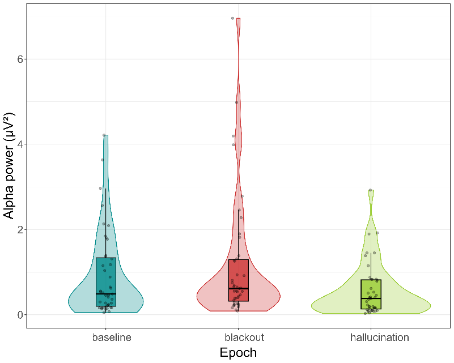

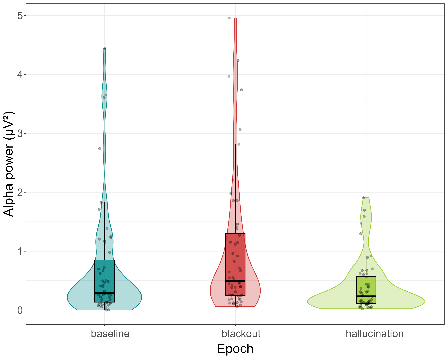


Experiment 1

Experiment 3

Experiment 2

†

†

*

*

*

*

baseline decay hallucination

**
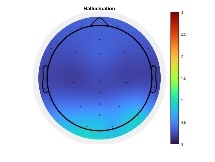

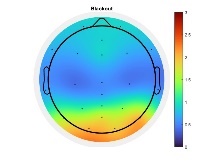

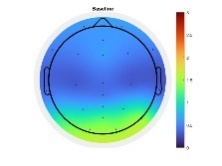

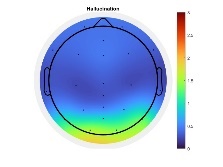

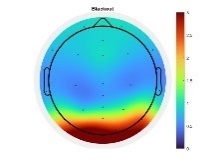

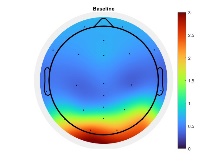
**
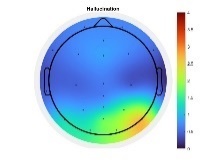
**
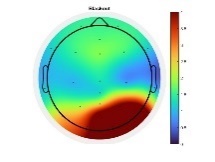

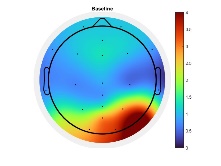
**

*Figure 1.* Violin plots of alpha power in the different epochs of interest and corresponding topographical plots of alpha power for Experiment 1, Experiment 2, and Experiment 3, showing increased alpha power for decay epochs relative to baseline and hallucination epochs. Figure licensed under CC BY 4.0 by the authors. Retrieved from https://doi.org/10.6084/m9.figshare.28451510

Experiment 3

Experiment 2

Experiment 1

*

*

*

*

*
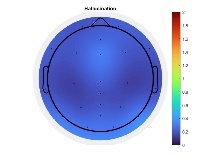
*
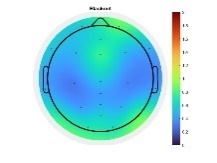

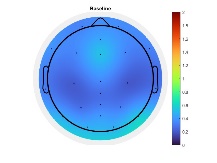

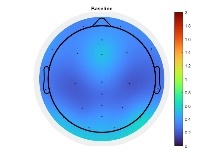

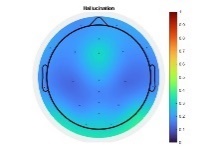

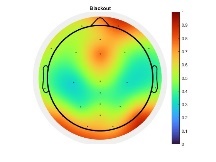
*
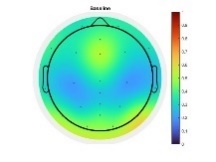

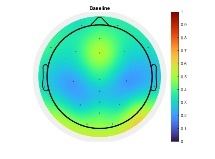

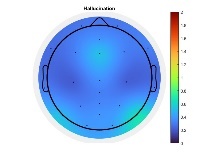

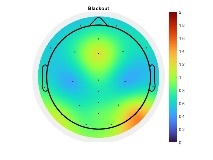
*
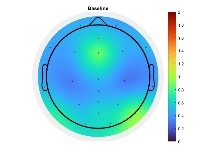

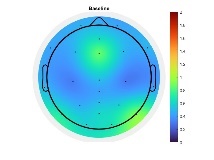

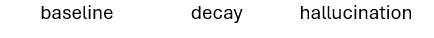

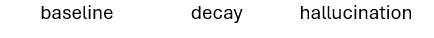

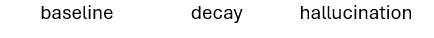

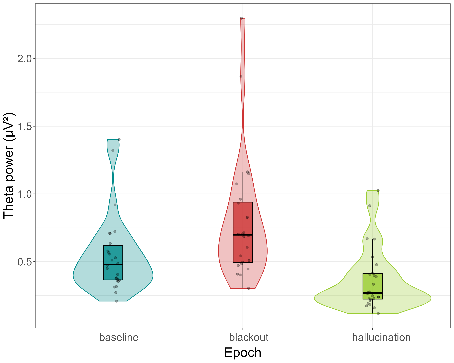

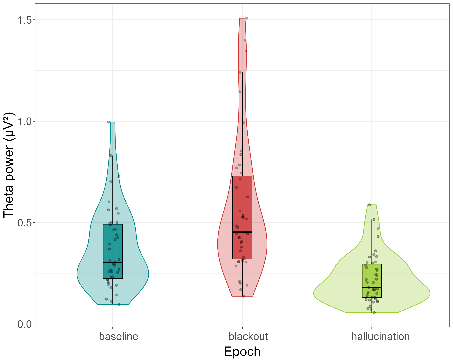

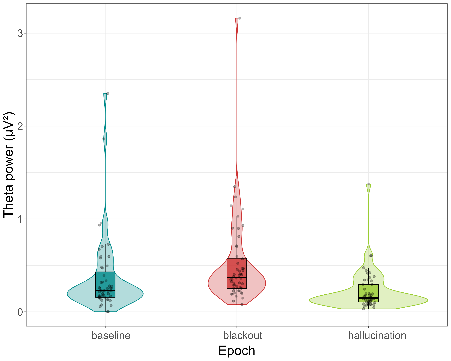


*

*

*

*

*

*Figure 2.* Violin plots of theta power in the different epochs of interest and corresponding topographical plots of theta power for Experiment 1, Experiment 2, and Experiment 3, showing increased theta power for decay epochs relative to baseline and hallucination epochs. Figure licensed under CC BY 4.0 by the authors. Retrieved from https://doi.org/10.6084/m9.figshare.28451513
